# Microfluidic Applications in Prostate Cancer Research

**DOI:** 10.3390/mi15101195

**Published:** 2024-09-27

**Authors:** Kailie Szewczyk, Linan Jiang, Hunain Khawaja, Cindy K. Miranti, Yitshak Zohar

**Affiliations:** 1 Department of Aerospace and Mechanical Engineering, University of Arizona, Tucson, AZ 85721, USA; kailieszewczyk@arizona.edu (K.S.); jiangl@arizona.edu (L.J.); 2 Cancer Biology Graduate Interdisciplinary Program, University of Arizona, Tucson, AZ 85724, USA; hkhawaja@arizona.edu; 3 Department of Molecular and Cellular Biology, University of Arizona, Tucson, AZ 85721, USA; cmiranti@arizona.edu; 4 University of Arizona Cancer Center, University of Arizona, Tucson, AZ 85724, USA

**Keywords:** microfluidics, prostate cancer, metastasis, dormancy, detection and therapy

## Abstract

Prostate cancer is a disease in which cells in the prostate, a gland in the male reproductive system below the bladder, grow out of control and, among men, it is the second-most frequently diagnosed cancer (other than skin cancer). In recent years, prostate cancer death rate has stabilized and, currently, it is the second-most frequent cause of cancer death in men (after lung cancer). Most deaths occur due to metastasis, as cancer cells from the original tumor establish secondary tumors in distant organs. For a long time, classical cell cultures and animal models have been utilized in basic and applied scientific research, including clinical applications for many diseases, such as prostate cancer, since no better alternatives were available. Although helpful in dissecting cellular mechanisms, these models are poor predictors of physiological behavior mainly because of the lack of appropriate microenvironments. Microfluidics has emerged in the last two decades as a technology that could lead to a paradigm shift in life sciences and, in particular, controlling cancer. Microfluidic systems, such as organ-on-chips, have been assembled to mimic the critical functions of human organs. These microphysiological systems enable the long-term maintenance of cellular co-cultures in vitro to reconstitute in vivo tissue-level microenvironments, bridging the gap between traditional cell cultures and animal models. Several reviews on microfluidics for prostate cancer studies have been published focusing on technology advancement and disease progression. As metastatic castration-resistant prostate cancer remains a clinically challenging late-stage cancer, with no curative treatments, we expanded this review to cover recent microfluidic applications related to prostate cancer research. The review includes discussions of the roles of microfluidics in modeling the human prostate, prostate cancer initiation and development, as well as prostate cancer detection and therapy, highlighting potentially major contributions of microfluidics in the continuous march toward eradicating prostate cancer.

## 1. Introduction

Prostate cancer is one of the most common cancers in American men, second to skin cancer, and it is the second leading cause of cancer death, behind lung cancer. For the year 2023, the American Cancer Society estimated 288,300 new cases of prostate cancer, with 34,700 deaths in the United States [1]. Like other types of cancer, prostate cancer tumors are not necessarily life-threatening as they may recede into a dormant state of quiescence [2]. Under normal conditions, stem cells differentiate into mature organ/tissue-specific cells steadily, but their growth can be accelerated during wound healing or malignancy like cancer. Proliferation of carcinomas has been linked to the presence of cancer stem cells (CSCs), while prostate CSCs (PCSCs) have been implicated in resistance to therapy [3]. Prostate cancer may lead to deadly complications if it turns metastatic as tumor cells spread from the prostate, through lymph or blood vessels, to other organs within the body, such as the bones, lungs, and liver [4]. Metastasis in prostate cancer is a multi-step process that involves extensive epithelium–stroma interactions in the tumor microenvironment, which includes local invasion, intravasation, extravasation, and colonization. Invading cancer cells intravasate the circulatory system by crossing the endothelial barrier of lymph or blood vessels, turning into circulating tumor cells (CTCs) capable of spreading far from the primary site. Prostate CTCs have been found to extravasate the circulatory system primarily to the bone, where they create secondary tumors that could be fatal. The metastatic process, requiring a coordination of multiple events, is rather heterogeneous. Most disseminated prostate cancer cells remain dormant, often for many years, as either individual cells or small cell clusters, but a small subset will develop into macro-metastatic lesions [5]. While cancer cells can leave tumors as cell clusters or single cells, distinct cancer cell clones show cooperative behavior promoting their mutual survival and metastatic ability. Polyclonal metastatic seeding has been documented in patients with prostate cancer and, in experimental models, polyclonal clusters of CTCs established metastases more efficiently than single cells [6]. The molecular mechanisms underlying heterogeneous metastatic efficiency of prostate cancer cells are poorly understood. Each phase of prostate cancer development, from tumor initiation to distant organ colonization, is under intense investigation, requiring adequate models to better understand the mechanistic determinants of the disease.

Prostate cancer research, like any other study in human biology, relies heavily on model systems. The scope and limitations of every project are directly affected by the selected model, which dictates the cost, timeframe, and interpretation of experimental results. In vitro mono-layered cell cultures and in vivo animal models have, for generations, been the two major tools in biological research, each with advantages and disadvantages [7]. Cell culture experiments are highly scalable; more experiments can be performed more rapidly than using animals, but animal models allow for testing on intact physiological systems of many cell types simultaneously. Although cell cultures have been useful for investigating cellular mechanisms, they poorly model physiological behavior [8], mainly because of the lack of appropriate microenvironments with proper crosstalk among various cell types [9]. Cells are embedded in extracellular matrices (ECMs), which provide mechanical support, regulate mechanical properties, as well as communicate extracellular signals to the cells. The ECM is present within all tissues and organs, providing scaffolding for the cells while initiating biochemical and biophysical cues needed for tissue homeostasis [10]. Cell cultures further suffer from a lack of interstitial flow, playing an important role in the biology of tissues. Interstitial fluid is in constant flow estimated to make up about 20% of the body’s mass, and, in living tissues, it is linked, with lymphatic drainage returning leaked plasma to the blood circulation [11]. Interstitial flow supplies fresh nutrients to cells while removing waste products and continuously affecting cell–cell interactions. On the other hand, the physiology of animal models is different from that of humans and, consequently, they have less than 8% successful translation to therapies in some cancer trials [12]. These shortcomings underscore the need to develop new in vitro systems that better mimic the in vivo human physiology in an effort to hasten biomedical innovation, including prostate cancer research.

Microfluidics, the science and technology dealing with microscopic quantities of fluid, is emerging as a promising candidate to provide solutions for many challenges in basic and applied biology. A testament to the rapid expansion of microfluidic technology specifically for biological applications, including cancer research, is the numerous review articles published just in the last few years [13,14,15,16,17,18]. As an extension of micromachining techniques developed for the electronics industry, microfluidic technology enables the fabrication of microfluidic devices (chips) with a micrometer scale feature size for processing micro-to-picolitre volumes of fluid on-chip at a time. Using such small volumes results in reduction of sample and reagent consumption per assay, making the analysis faster, more affordable, and environmentally friendlier than other methods. Fully integrated microfluidic systems designed for autonomous analysis involve five core components: sample introduction, movement, purification, detection, and analysis [19]. In comparison with conventional cell cultures and animal models, microfluidic systems enable greater reproducibility and superior control over the cellular microenvironment [20]. Coupled with the small scale of equipment used, the high level of automation, and the relatively cheap production, the application of microfluidics in medicine is continuously increasing [21], with significant promise in cancer diagnosis and management [22]. Microfluidics has emerged in the last decade as a new field that could inspire a paradigm shift in biomedical research including prostate cancer. With the development of the organs-on-chips concept, microfluidic systems have been advanced to recapitulate the critical functions of many human organs [23]. These microphysiological systems (MPSs) enable the establishment of 3D cell co-cultures, aiming to mimic in vivo microenvironments of many human organs, such as the lung and liver [24]. This has culminated with the introduction of human-body-on-a-chip, where MPSs representing critical organs were connected and controlled [25]. Utilizing microfluidic technology in urology has been reviewed in some articles discussing prostate cancer [26,27], and particularly those assessing castration-resistant prostate cancer [28]. Aware that prostate cancer can relapse long time after post-treatment and disease-free survival, the discussion in this article has been expanded to include applications of microfluidic systems in several important facets of prostate cancer. Some microfluidic platforms originally utilized for studying other human cancers are also discussed as they could readily be adapted for prostate by incorporating the proper cell types. As summarized in Table 1, we review here microfluidic systems recently reported for investigating numerous aspects of prostate cancer, from primary tumor initiation to colonization of remote organs, as well as detection and therapy for clinical applications, showcasing the transformative impact of microfluidics. Following a brief introduction, the human prostate physiology is presented, including microfluidic in vivo and vitro modeling of the human prostate. This is followed by a discussion of the contribution of microfluidics to research of prostate cancer development, from its initiation to metastasis culminating in organ colonization. Then, various microfluidic approaches for prostate cancer detection are described based on patient blood and urine samples. Finally, several microfluidic aspects related to prostate cancer therapy, particularly drug discovery and screening, as well as well its treatment and management, are considered. Undoubtedly, the contribution of microfluidics is poised to increase in the future in the persistent effort to achieve the long-term goal of eradicating prostate cancer.

## 2. Human Prostate Modeling

The human prostate is a small organ in males surrounding the urethra just below the bladder. It is a sex accessory gland that produces and secretes fluids nourishing and protecting sperm and, thereby, significantly enhancing male fertility. The prostate is made up of a system of branching ducts and acini, where the fluid is secreted, comprising a bistratified epithelium surrounded by a fibromuscular stroma with a basement membrane (BM) separating the epithelium from the stroma [29]. A mature prostate epithelium contains several distinct cell types that differ in their morphology. Luminal cells are tall columnar secretory epithelial cells that express cytokeratins (CK8 and CK18) and secrete proteins such as prostate-specific antigen (PSA); all luminal cells express high levels of androgen receptor (AR) and AR-regulated proteins. Non-secretory basal cells line the BM under the luminal layer; they express certain types of integrins (e.g., α6 and β1), cytokeratins (e.g., CK5 and CK14), and low or undetectable levels of AR. Occasional intermediate cells co-expressing luminal and basal markers, as well as additional markers, such as CK19, are present within the basal layer, and it remains unclear whether intermediate cells represent a functionally distinct cell group. Rare neuroendocrine cells are basally localized expressing secreted neuropeptides and other hormones, often displaying a dendritic-like shape that contacts the glandular lumen. Despite the existence of several cell populations in the epithelium, the basal cell layer appears to be continuous in histological sections of the human prostate. The stromal compartment of the prostate comprises a few differentiated cell types as well. Cells of the embryonic urogenital sinus mesenchyme (UGM) form a layer of smooth muscle, which lines the epithelium and exhibits a contractile characteristic to aid the expulsion of the prostatic fluid into the ejaculate. The adult prostate stroma contains a population of mature fibroblasts that secrete extracellular matrix (ECM) consisting of fibrillary proteins, glycoproteins, and proteoglycans to form a structural network and mediate growth factor signaling. Other components of the stroma include blood vessels, lymphatics, nerve, and immune cells, which have been implicated in the stem cell regulation as well as tumorigenesis within the prostate. Human prostate ducts are held within a continuous thick mass of fibromuscular stroma, where stromal and epithelial cells are present in equal numbers. The prostate is apparently prone to oncogenic mutations at a frequency significantly higher than that of other male secondary sexual tissues, such as the seminal vesicles. Approximately 15% of men will be diagnosed with prostate cancer during their lifetime [30] and, not surprisingly, the disease has been the focus of intense research to understand its pathobiology and to seek efficient therapy [31]. Malignancy, including that of the prostate, has been hypothesized to occur due to re-awakening of developmental processes taking place during organogenesis. Indeed, some studies have demonstrated key similarities in gene expression programs between prostate organogenesis and cancer, lending credence to the need for gaining a comprehensive understanding of the fundamental mechanisms behind prostate development [29].

### 2.1. In Vivo Animal Models of the Human Prostate

The prostate arises from epithelial buds emerging from the embryonic urogenital sinus (UGS), and its development has been studied in many mammalian species. While interest in prostate biology is centered on the human organ, the species prostate is attractive for modeling human diseases. The most detailed description of prostatic development has been reported for mouse and rat, and prostatic development in humans is still under-reported [32]. Prostate development follows a common pattern and, in all species examined, including human, it depends on the actions of androgens to induce and support ductal branching morphogenesis of the epithelial buds. The developmental process is remarkably similar in all species, and species-specific details of prostatic development and anatomy have been noted. The anatomy of the human prostate is significantly different from that seen in laboratory animals. Furthermore, the gross structure of the adult prostate found in most mammals differs considerably between species. The human prostate has a compact zonal anatomy immediately surrounding the urethra and below the urinary bladder, whereas rodents have a lobular prostate with lobes radiating away from the urethra. The disease profile of the human and rodent prostate is also very different; the human prostate is the site of several diseases but, in contrast, the rodent prostate has few naturally occurring ones [33]. Animal models have proven to be useful in understanding underlying mechanisms of many human diseases. Rodents can be used to model certain aspects of human diseases, such as prostate cancer, but not the whole disease profile. For example, using a rat model, two growth factors (IGF-1 and VEGF) were implicated as critical regulators in mediating epithelial–stromal interactions in hormone-induced prostate carcinogenesis [34]. However, the structure of the human and rodent prostates is so different that the extrapolation of data between species is very limited. A clear understanding of the differences in the structure of human and animal prostates is important in assessing the results of animal studies and applying them to humans.

### 2.2. In Vitro Models of the Human Prostate

The most prominent cells in the human prostate epithelium are high columnar luminal cells with secretory activity, and stretched basal cells adhering to the basement membrane. Two opposing hypotheses have been suggested to explain the cellular homeostasis of the human prostate epithelium [35]. While observations of proliferative activity in adult rat studies have indicated self-renewal of both basal and luminal cells, others have postulated that the entire prostate epithelium is derived from stem cell populations residing within the basal cell layer. These cell populations amplify the prostate epithelium by transient proliferation, eventually resulting in terminally differentiated cells that undergo apoptosis. Regardless, like other stratified epithelia, prostate basal cells are mitotic and adhere to the basement membrane, giving rise to terminally differentiated secretory luminal cells [36]. However, unlike other epithelia, prostate epithelial differentiation is regulated by androgen signaling [37,38]. The androgen receptor (AR) is a nuclear transcription factor activated in response to the steroid hormone androgen, which is expressed in the differentiated secretory cells but not in the basal cells [39]. Using culture dishes, an in vitro differentiation model of human prostate epithelium composed of stratified cells that recapitulates many in vivo characteristics of epithelial basal and secretory luminal cells, including AR-dependent differentiation and function, was established [40]. This model seems amenable to functional and biochemical analysis; however, the clarification of the stroma’s role in prostate epithelial differentiation and survival was hampered by the lack of stroma.

Two-dimensional cell culture models, popular due to their simplicity, have been extremely useful in delineating intracellular mechanics, shedding light on the fundamentals of cell biology. However, they have proven to be poor predictors of physiological responses since tissues and organs are multicellular structures organized in 3D microenvironments, where cells are exposed to a far greater number of cues. Indeed, the establishment of 3D cell cultures has led to the realization that cell–cell and cell–matrix interactions, as well as cell morphology and differentiation, differ drastically from those in culture dishes [41]. The in vivo microenvironment is a bundled network of macromolecules and minerals containing soluble signals known as the extracellular matrix (ECM). Cell fate and function in a tissue depend on several factors: (i) physical signals due to interaction with the ECM serving as structural support, (ii) secreted soluble signals like cytokines and chemokines, and (iii) signals due to direct cell–cell interactions. Another often-ignored factor is the interstitial fluid flowing over cells and, thereby, influencing multiple cellular properties and processes [42]. Interstitial flow also exerts mechanical shear stress affecting cells morphology, growth, and differentiation [43]. These factors must clearly be incorporated in efforts to establish successful in vitro models of human organ tissues. Indeed, new synthetic biomaterials are being developed specifically for use as 3D extracellular microenvironments to mimic the regulatory characteristics of natural ECMs and ECM-bound growth factors [44]. Classification of a cellular system as a 3D model though requires some caution since it has been loosely used to characterize both cell monolayers in 3D configurations and co-cultures of planar (2D) multicellular layers. Nevertheless, both approaches promote cell differentiation and tissue organization levels not possible in standard cell culture models. Furthermore, leveraging recent advances in 3D cell cultures with microfluidic technology allowed the creation of microphysiological systems (MPSs) that reconstitute the tissue differentiation, tissue–tissue interfaces, chemical gradients, and physical microenvironments of human organs [45]. These MPSs, often called ‘organs-on-chips’, permit the study of organ-specific physiology as has been demonstrated for the lung, liver, kidney, gut, skin, brain, and heart [46]. Furthermore, given the large amount of data produced by the high parallelization of MPSs, deep learning is emerging as a promising tool to analyze ‘big data’ for personalized medicine [47]. However, despite the high prevalence of life-threatening diseases and cancers that affect the exocrine glands, there are few reports of MPSs designed for the research of ductal/acinar systems such as the human breast and prostate.

An Intriguing microfluidic method to fabricate single straight circular channels was presented to mimic epithelial-lined ductal systems of glandular tissues for studying adherent cells while enabling manipulation of the cellular microenvironment [48]. The technique is based on using glass capillaries with corresponding needles to serve as a mold for polydimethylsiloxane (PDMS)- or hydrogel-based devices. The system biocompatibility was demonstrated using various epithelial cell lines that grew stably inside the microchannels. Epithelial cells in 3D culture reproduced essential structural features of glandular epithelium in vivo, with the presence of a centrally hollow lumen and the polarization of cells surrounding it. The influence of the 3D microenvironment in the tubes on the epithelial cell morphology and proliferation was further investigated by coating the microtubes either with a layer-by-layer assembled polyelectrolytes (PE) film or with Matrigel. The PE film provided a tunable surface environment for external manipulation of cells, whereas the Matrigel was typically used to mimic the complex extracellular environment found in many tissues. This microfluidic system enabled the formation and access to an artificial lumen, enabling the investigation of cell morphogenesis and events occurring during lumen population by cells as observed in cancer development.

Clearly, it is not feasible to replicate every detail of the adult prostate in vitro since it is a highly complex system containing multiple cell types with intricate interactions among them. Instead, the aim is to simplify the biomimetic system, allowing the recapitulation of only the dominant structural and functional features of the human epithelium–stroma interface, which is the fundamental component of a living prostate. The major obstacle for realizing 3D co-cultures of different types of cells in dishes and transwells is perhaps due to the mixing of incompatible specific media required for maintaining different cell types. Therefore, following the organ-on-a-chip concept, a microfluidic system was developed to provide a relevant in vitro model mimicking the critical functions of a human prostate gland observed in vivo [49]. The system featured a microfluidic bilayer device, consisting of two microchannels stacked on top of each other and separated by a porous membrane, designed for long-term co-cultivation of human epithelial and stromal cells. The porous separation membrane provided an anchoring scaffold for culturing the two cell types on its opposite surfaces, and its pore size was selected to allow cell signaling via molecular diffusion but not cell crossing between the two channels. This MPS involved controlled the flow of culture media at a rate adjusted to maximize communication between the two compartments and to minimize mixing of the media. The established human epithelium–stroma interface was then utilized to simulate the functional development of the in vivo human prostate gland. The PDMS microfluidic device was transparent, permitting high-resolution, bright-field, and fluorescence imaging. Successful differentiation of basal epithelial into luminal secretory cells was biochemically determined by immunostaining known differentiation biomarkers, particularly the androgen receptor expression level. Morphological changes were also observed, where gland-like mounds appeared with relatively empty centers reminiscent of prostatic glandular acini structures. Thus, this prostate MPS can facilitate the direct evaluation of paracrine and endocrine crosstalk between these two cell cultures, as well as studies associated with normal vs. disease-related events as in prostate cancer.

## 3. Prostate Cancer Development

Major challenges in cancer research remain the establishment of reliable tumor microenvironment in vitro models and anticancer drugs screening techniques to shed light on the nature of cancer and discover new remedial therapies. It has been widely recognized that limitations of classical cell cultures and animal models have severely hindered the translation of basic research results to clinical applications. Consequently, various three-dimensional (3D) biomimetic models have emerged that can fill a technical gap between conventional 2D approaches and preclinical in vivo models [50] such as patient-derived explants (PDEs); these ex vivo cultures of freshly resected prostate tumor specimens, obtained from surgery, provided a high-throughput and cost-effective model retaining the native tissue architecture, microenvironment, cell viability, and key oncogenic drivers [51]. Microfluidic technology, though, used to fabricate devices designed for cancer research, is considered as the most promising approach to meet the in vitro modeling demands. The continuous expansion of microfluidics, from cells to organs, has resulted in the realization of sophisticated MPSs, which can be utilized for exploring key features of tumor microenvironment such as angiogenesis, tumor–stromal interactions, and the extracellular matrix [52]. Indeed, critical structural and functional characteristics of the in vivo tumor microenvironment have been recapitulated successfully in vitro [53]. Here, recent advances in the application of microfluidics specifically to prostate cancer research are reviewed.

### 3.1. Prostate Carcinogenesis

The human prostate is the site of origin of three major diseases: (i) benign prostatic hyperplasia (BPH), a condition in which the prostate gland is enlarged; (ii) prostate cancer (PCa); and (iii) prostatitis, which is inflammation of the prostate gland [33]. Despite the accumulation of massive amount of data acquired to decipher the genetics of prostate cancer risk, the molecular mechanisms driving prostate cancer development and progression are still poorly understood. In contrast, significant information has been obtained about the morphological and molecular changes that take place during prostate cancer initiation and progression [54]. Prostate carcinogenesis and tumor progression are driven by complex interplay between inflammation, immunity, and tumor genomics. There is mounting evidence that multiple cell types can act not only as progenitors for fully differentiated secretory luminal cells but also as cells of origin for prostate tumors, which may relate to the genotypic heterogeneity in prostate cancer [55]. Therefore, it seems plausible that any progenitor cell that could give rise to the luminal lineage could also respond to oncogenic transformation by generating malignant luminal progeny. However, it is yet to be determined whether the cell of origin influences the fate of the tumor (aggressive vs. indolent) or whether this is determined by genetic alterations or the tumor microenvironment. During prostate tumorigenesis, oncogenic signaling pathways promote the progression of androgen-dependent carcinomas to castration-resistant prostate cancer (CRPC), the major contributing factor in PCa fatalities.

#### 3.1.1. Adenocarcinoma of the Prostate (Carcinoma In Situ)

**Figure 1:**
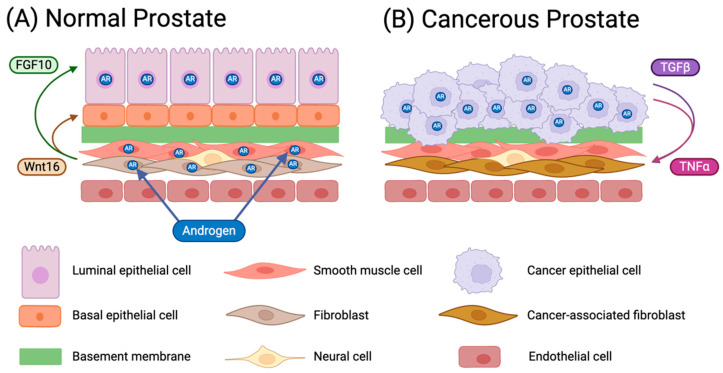
A comparison between normal and cancerous prostate: (**A**) In the normal prostate, the tissue structure is well organized, with stratified basal and luminal cell epithelium separated by a basement membrane from smooth muscle and fibroblast cell stroma; it is maintained by stromal AR via secretion of Wnt16 and FGF10 (luminal, smooth muscle, and fibroblast cells express AR). (**B**) In the cancerous prostate, the glandular organization is lost, with a loss of basal cells and decomposed luminal ductal structure due to the suppression of stromal–AR expression and the conversion of stromal cells to cancer-associated fibroblasts (CAFs) by tumor secretion of TNFα and TGFβ1 (tumor cells retain AR expression).

There is a strong link between prostate cancer and inflammation, as chronic inflammation causes proliferative inflammatory atrophy that may progress to the precursor of prostate cancer known as prostatic intraepithelial neoplasia (PIN) [54]. Prostate carcinoma mainly involves an accumulation of cancerous epithelial cells, but non-epithelial cells also play a very important role in prostate cancer development; significant changes in both the epithelium and stroma take place during prostate cancer initiation and progression. Enlargement of the prostate due to an increase in the reproduction rate of epithelial cells, particularly in older individuals, results in a hyperplastic prostate epithelium. This condition, known as benign prostatic hyperplasia (BPH), is not considered to be a precursor to prostate carcinoma. The most common type of prostate cancer is (acinar) adenocarcinoma, originating from secreting cells that line the prostate gland, while ductal adenocarcinoma is relatively rare, originating from cells lining the tubes of the prostate gland [56,57,58]. Some anatomical differences between a normal and a cancerous prostate are illustrated in Figure 1. The normal prostate features clearly defined ducts: each lined by a well-differentiated epithelium composed of a layer of luminal cells, exposed to the ductal lumen, on top of an organized layer of basal cells that are attached to a BM (Figure 1A); normal basal-to-luminal cell differentiation is regulated by stromal AR. The epithelium is surrounded by the stroma, on the other side of the BM, with fibroblast and smooth muscle cells as the main constituents. In contrast, in the cancerous prostate, the morphology is highly irregular with largely collapsed ducts. The clear distinction between basal and luminal cells is lost in the overgrown epithelium, and the stroma alignment to the epithelium is compromised with a degraded BM (Figure 1B); the stromal AR is lost accompanied by the cell conversion to cancer-associated fibroblasts (CAFs). Upon transformation, either basal or luminal cells can invade across the basement membrane and into the surrounding stroma or ECM, forming an invasive ductal carcinoma. Further alterations in gene expression can then lead to a metastatic cascade, whereupon cells can travel to distant organs and colonize new sites of growth and invasion.

#### 3.1.2. Monoculture Models

Cells in tissues are embedded in the ECM composed of polysaccharides and proteins, and their relative amount is tissue-specific. The ECM is not just structural but also instructive playing a role in regulating cellular behavior and affecting cell proliferation, shape, function, migration, and survival. However, in vitro models of tissues/organs require culturing cells on surfaces, and weak cell adhesion to the culture surfaces is a known problem addressed by various surface-coating protocols. The response of PC3 cancerous epithelial prostate cells growing on polyelectrolyte (PE)-coated surfaces was investigated [59]. The proliferation rate of PC3 cells decreased on PAH (poly(allylamine hydrochloride))-terminated and slightly increased on PSS (poly(sodium 4-styrenesulphonate))-terminated coating; only PAH prevented clustering of PC3 as compared to fibronectin or PSS coatings. Surface coating therefore can be used as a tool to preferentially modulate the responses of prostate tumor cells. Weak surface attachment of cells, e.g., LNCaP, has impeded their application and analysis. For optimal culture conditions, different coating reagents were tested [60]. Reagents like fibronectin improved LNCaP cells adherence, provoking cell morphology alterations; in contrast, collagen type IV and laminin promoted cell aggregation, affecting cell morphology, but did not improve cell adherence. Hence, in utilizing microfluidic devices, coating protocols should be adjusted specifically for the selected substrate material and cell type combination that can significantly alter experimental outcomes. Passaging is invariably involved in cell culture maintenance for research, and trypsin is commonly used for repeated passages of cells. However, trypsin digests the coatings on bio-functionalized surfaces, which could limit the number of cell passages compromising the system performance [61]. Detachment of adhering individual cells under hydrodynamic loads, on the other hand, results in significant cell deformation that may alter their phenotype [62]. To address this issue, a microfluidic chip has been fabricated for the continuous culture and passage of PC3 prostate cancer cells [63]. Flexible diaphragms were used to apply hydrodynamic shear forces, detaching a fraction of the adhering cancer cells from the surface for output while leaving the rest for reuse in subsequent cultures. As cell mechanical properties were suggested as markers for metastatic potential, a morphological rheological microfluidic method was applied for the high-throughput mechanical phenotyping of androgen-sensitive and non-sensitive human prostate cancer cell lines [64]. The velocity and deformation of cells, passing through a constriction microchannel, were analyzed. The elastic modulus of an androgen-sensitive prostate cancer cell line (LNCaP) was found to be smaller than the modulus of two androgen-non-sensitive prostate cancer cell lines (PC3 and DU145). The loss of cell polarity was implicated as an early event in epithelial carcinogenesis; thus, an imaging method was presented to analyze the polarity in RWPE-1 normal and WPE1-NB26 cancerous human prostate epithelial cells, which form polarized acini with lumen in 3D cell cultures [65]. The methodology enables the analysis of epithelial tissue morphogenesis in a large field of view, elucidating the regulation of growth, morphogenesis, and differentiation in normal and cancerous human prostate cells. To enhance the patient-derived conditionally reprogrammed cells (CRCs) application for prostate cancer, a 3D culture protocol was reported, enabling a rapid luminal cell differentiation in normal and in tumor-derived prostate epithelial cells [66]. A filter insert-based technique was utilized for culturing and differentiating normal and malignant prostate CRCs, including cell collection and processing, e.g., immunohistochemical and immunofluorescent staining. This culture method, with primary CRC lines, provided a model system that can facilitate the development of “biobanks” of patient derived cell lines for biospecimen-based prostate research.

These examples of microfluidic devices and techniques offer robust prostate cancer cell culture and output technology, requiring less human intervention and reducing contamination problems, with label-free biomarkers to determine cell physiology or pathology fate. In all these microfluidic platforms, developed to address specific issues in prostate cancer research, only single tumor cell cultures were used. However, it is well established that multi-cell types are present in the tumor microenvironment, particularly the stroma, playing an important role in cancer development and progression. Hence, microphysiological systems aspiring to serve as in vitro models for human prostate cancer must incorporate at least two, and most likely more, different prostate cell types to gain traction with researchers in the field.

#### 3.1.3. Co-Culture Models

Several articles have focused on the current understanding that tumor development and progression is determined not only by the tumor cells but also by the tumor microenvironment; this includes an intricate network of different cell types, e.g., stromal cells, immune cells, and endothelial cells, interacting via the extracellular matrix and soluble factors such as cytokines, chemokines, and metabolites. For example, transformed epithelium was incapable of maintaining normal differentiation of adjacent muscle; in turn, abnormal stroma resulting from the dedifferentiation or reduction of smooth muscle cells (SMCs) could lead to loss of stromal control over epithelial proliferation and differentiation [67]. Consequently, a loss of differentiation in both epithelium and stromal SMCs may be critically involved in hormone-induced prostate carcinogenesis. The role of different stromal components in prostate cancer, as well as their potential prognostic and therapeutic significance, have been discussed [68]. Cancer-associated fibroblasts (CAFs) were found to have a crucial role in the proliferation, invasiveness, metastasis, and angiogenesis of prostate cancer [69], while mesenchymal stroma/stem cells (MSCs) were reported to play an important role during carcinogenesis exhibiting different types of intercellular communication [70]. TGF-β1 was identified as a crucial molecule for inducing MSC recruitment to PCa cells and tumor stroma components, while PCa- and tumor stroma-secreted TGF-β1 were important for inducing MSC trans-differentiation into carcinoma-associated fibroblast (CAF)-like cells [71]. Thus, tumor stromal cells have been implicated in supplying signals that may lead to aggressive phenotypes of carcinoma cells culminating in metastasis. Gene expression profiling was performed to obtain epithelial and stromal transcriptional maps of prostate cancer initiation and progression potential [72], and the data strongly suggest that tumor cells and cells in the cancer microenvironment are co-dependent and co-evolving as the microenvironment influences prostate cancer initiation, maintenance, and metastasis.

In vitro cell culture models simulating tissue or tumor microenvironment are clearly necessary tools to delineate epithelial–stromal interactions, including paracrine function. To this end, a 3D co-culture model in double-layered alginate hydrogel microspheres was developed, incorporating prostate cancer epithelial and stromal cells in separate compartments of the microspheres [73]. Measurements of shaded component of E-cadherin (sE-cad) in the conditioned media, demonstrating the amount of sE-cad dependency on epithelial-stromal interaction, suggested that the model can be used to study cell–cell paracrine interaction. However, reconstitution of complex microenvironments in microfluidic devices has emerged as a fundamental tenet for their superior performance compared to all other alternatives. Accordingly, several microfluidic systems have been developed either specifically or that are easily adopted for prostate cancer research. Interactions between cancer and immune system were investigated using an on-chip model, where melanoma and immune cells were co-cultured in a PDMS platform, and the results confirmed in vivo observations, highlighting the value of on-chip co-cultures to investigate interactions between cancer cells and the immune system [74]. A microfluidic chip-based tumor tissue model was presented integrating 3D tumor spheroids (TSs) with CAF in proximity, within a hydrogel scaffold, to establish a ‘more clinically relevant’ tumor model [75]. Enhanced growth of human colorectal TSs was observed when co-cultured with fibroblasts, while the fibroblasts were activated under co-culture conditions. Aiming to understand combinatorial effects of multiple biochemical and microenvironmental factors, a microfluidic system consisting of microchamber arrays embedded in a collagen hydrogel with tunable biochemical was constructed to investigate the interactions between invasive breast cancer cells and stromal cells [76]. The hollow microchambers in collagen provided an environment similar to that in vivo, regulating collective cellular dynamics and behavior, and the microfluidic channels surrounding the collagen microchamber arrays allowed the generation of complex concentration gradients of specific biological molecules or drugs. Specifically for prostate cancer research, a microfluidic-based model of the human prostate gland was reported for co-culturing epithelial and stromal cells [77]. Various combinations of human prostate epithelium and stroma were co-cultivated, and the interactions between the two types of tissues were investigated. While normal epithelium differentiation was shown to be induced and regulated by normal stroma, normal stroma assumed a cancer-associated fibroblast phenotype when co-cultivated with cancerous epithelium. Subsequently, the microfluidic model was further developed to establish a microfluidic-based human prostate-cancer-on-chip (PCoC) [78]. Human prostate cancer and stromal fibroblast cells were co-cultivated in two channels separated by a porous membrane under culture medium flow. The established microenvironment enabled soluble signaling factors, secreted by each culture, to diffuse through the membrane pores affecting the adjacent culture. The tumor cells downregulated androgen receptor (AR) expression and induced CAF biomarkers in stromal fibroblasts. Tumor invasion into the stroma was also investigated in devices featuring a serpentine channel, with orthogonal channel segments, overlaying a straight channel. Both tumor cells and stromal CAFs crossed over into their neighboring channel, through the separation membrane, as the role of the stroma seemed to be proactive in promoting cell invasion. Spatiotemporal analysis of the tumor–stroma dynamic interactions in the PCoC model suggested that the in vivo tumor microenvironment of prostate cancer could be replicated faithfully, thus providing a clinically relevant in vitro human prostate tumor model, with the porous membrane representing the in vivo basement membrane.

### 3.2. Prostate Cancer Metastasis

**Figure 2:**
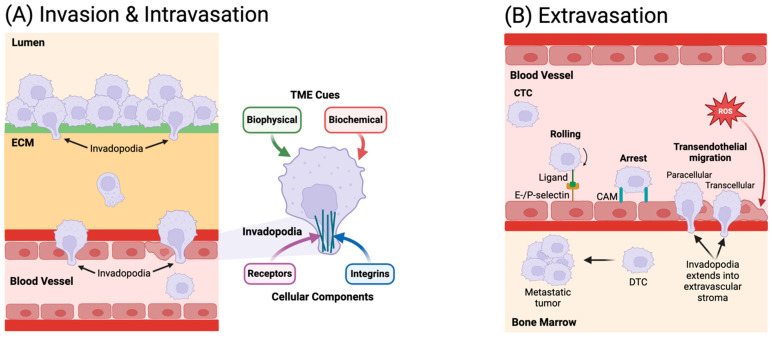
Invasion, migration, and intravasation in prostate cancer: (**A**) Tumor cells undergoing EMT form invadopodia in the cellular cytoskeleton, acquiring a migratory phenotype, which enable the degradation of basement membranes and extracellular matrix using metallo proteases; invadopodia are involved in directional migration when crossing the ECM and, upon reaching blood vessels, tumor cells use invadopodia again to penetrate the endothelium entering the bloodstream as CTCs. Formation and activity of invadopodia, actin-rich protrusions of the plasma membrane, are regulated by biochemical and biophysical signals derived from the TME and mediated by membrane integrins and receptors. (**B**) Rolling of CTCs is initiated by ligands biding to selectins on activated endothelial cells at sites of inflammation or vessels of the bones, where E-selectin is expressed constitutively, followed by their firm adhesion to the endothelium via integrins binding to their endothelial counter-CAMs. Endothelial retraction is then mediated by cytokines stimulating the production of ROS, which may irreversibly damage the endothelium at sites of CTCs arrest. During intravascular migration, CTCs also form invadopodia that are extended across the endothelium into the extravascular stroma prior to either paracellular or transcellular extravasation. Upon successful trans-endothelial migration, either individual quiescent or dormant cluster cells form micrometastases in the host organ.

Metastasis is the process by which cancer cells leave their original/primary site to settle in a different/secondary site within the body, and it is a hallmark of cancer, differentiating it from benign tumors [79]. Most cancer deaths are caused by metastatic cancer damaging important organs and, consequently, the prognosis of prostate cancer is mainly determined by the presence or absence of metastases. While almost all cancers can metastasize, actual metastasis depends on many factors. Metastasis, in general, consists of a sequence of events including tumor expansion, invasion of cancer cells through the surrounding tissue, intravasation into the circulation, survival in the bloodstream, extravasation at distant sites, and subsequent organ colonization as sketched in Figure 2. There are three primary routes by which tumors can spread to distant organs: (i) through the circulatory (blood) system (hematogenous), (ii) through the lymphatic system, and (iii) through the body wall into the abdominal and chest cavities (transcoelomic) [80]. Many cancer cells die while traveling through the blood and lymph vessels, but some may survive and stick to the vessel wall. These cells then move through the vessel wall into another body tissue where they may divide and form a metastatic tumor, and the most frequent sites of distant prostate cancer metastases are the bone, lung, and liver [81]. Chemokines have a critical role in cancer progression, and two chemokine ligand–receptor systems are common key mediators of tumor cell metastasis for several malignancies; the CXCL12–CXCR4 axis facilitates metastasis to distant organs, whereas the CCL21–CCR7 chemokine ligand–receptor pair favors metastasis to lymph nodes [82]. Some known mechanisms for prostate cancer lymph node metastasis, along with the involvement of lymphatic vessels, have been discussed [83]. A comprehensive understanding of prostate cancer metastasis undoubtedly has significant clinical implications in resolving prognostic or therapeutic issues for prostate cancer patients, and microfluidics has already been applied to investigate each stage of the metastatic cascade. Organ-on-a-chip models have emerged as powerful tools to research the TME and its impact on metastasis. Cancer-on-a-chip platforms, in particular, enable an in vitro investigation of the metastatic process in a stepwise manner with precise control and unprecedented detail by recapitulating and simplifying the in vivo tumor microenvironment [84].

#### 3.2.1. Invasion

Prostatic intraepithelial neoplasia (PIN) is considered a precursor of invasive carcinoma, and early invasion in prostate cancer is characterized by disruption of the basal layer and is associated with the foci of high-grade PIN [85]. Invasion occurs when cancer cells detach from the tumor mass, migrating into the surrounding tissue (Figure 2A). As proliferation persists, cancer cells become confined and metabolically starved, promoting their epithelial-to-mesenchymal (EMT) transition, changing their morphology, and increasing their motility. EMT might be involved in the dedifferentiation program, leading to malignant carcinoma and giving rise to the dissemination of single carcinoma cells from the primary tumors. Several signal-transduction pathways have been identified for EMT, in which transcriptional repressors of the E-cadherin gene are activated, resulting in the loss of the epithelial phenotype [86]. The transcription factor zinc finger E-box binding homeobox 1 (ZEB1) has been identified as a key transcriptional regulator of EMT in prostate cancer, with its aberrant expression occurring partly in response to Insulin-like growth factor-I (IGF-I) IGF-I stimulation [87]. PRH/HHEX (proline-rich homeodomain protein/haematopoietically expressed homeobox protein) is another transcription factor controlling cell proliferation, differentiation, and migration in a variety of tissues. As hyper-phosphorylated PRH levels are elevated in benign prostatic hyperplasia, prostatic adenocarcinoma, and prostate cancer cell lines, increased phosphorylation of PRH in prostate cancer cells has been suggested to increase cell proliferation and cell migration/invasion [88]. The androgen receptor (AR), expressed in both prostate stromal and epithelial cells, has particularly been implicated in cell cancer adhesion and invasion. Androgens and androgen receptors modulate gene expression in cells, and the overexpression of several genes has been reported to be associated with prostate cancer invasion [89,90,91,92]. AR was suspected in promoting invasiveness of both androgen-dependent and independent prostate cancer; its expression affects EGFR-mediated signaling, and targeting EGFR in androgen-independent prostate cancer could inhibit tumor proliferation and invasion. Negative regulation of prostate cancer growth and invasion by stromal AR have also been reported, and Ets Variant Gene 1 (ETV1) was identified as a novel androgen-regulated gene [93,94,95,96]. Tumor cell migration is not merely genetically determined but also regulated by environmental signaling, including chemokines and neurotransmitters; the migration of prostate carcinoma cells (PC3) was shown to be enhanced in vitro by a stress-related neurotransmitter, norepinephrine, and could be inhibited by the beta-blocker propranolol [97]. One of the major mechanisms by which prostate cancer spreads out of the gland is perineural invasion (PNI), a process by which cancer cells wrap around nerves. A PNI in vitro coculture model was reported using a microchamber system, which consisted of human prostate cancer cells (DU145) and mouse ganglia/nerves embedded in Matrigel [98]. An increase in proliferation and a decrease in apoptosis was associated with nuclear translocation of the nuclear factor κB (NFκB), as cancer cells in a perineural location acquired growth advantage using an NFκB survival pathway. Gene microarray studies have also revealed Ets Variant Gene 1 (ETV1) as an AR-regulated gene that mediates prostate cancer cell invasion [99]. ETV1 mRNA and protein were upregulated in response to ligand-activated AR in androgen-dependent LNCaP cells whereas, in androgen-independent LNCaP cells, ETV1 expression was high and unresponsive to androgen. Disruption of ETV1 expression in both cell types significantly compromised their invasion capacity, suggesting an important role for ETV1 in prostate cancer invasion. During metastasis, all cancer types must migrate through crowded multicellular environments, often in confined spaces, continuously changing their phenotype. To dissect the cell deformation mechanism with its associated molecular events involved in tumor progression, a closed glass microfluidic chip was fabricated with 3D biomimetic polymer nanostructures [100]. Polymeric channels served as an EGF gradient generator to mimic proper environments for analyzing metastatic potential of cancer cells. PC3 cells were responsive to an in-channel EGF gradient suffering partial cytokinesis at the end of migration, followed by fusion of the fragments back into single-cell bodies. Softening and increased contractility of migrating cells, influenced by the microenvironment, have been proposed as biomarkers of metastatic progression; however, prostate cancer cells appear to deviate from these common cancer trends. The biophysical changes of prostate cancer cells, with increasing metastatic potential, were characterized as a function of the microenvironment stiffness [101]. Cells of various metastatic efficiencies exhibited unique biophysical responses differentially influenced by the substrate stiffness. To explore the matrix stiffness effect on EMT of prostate cancer cells (DU145 and PC3), the microenvironment was modeled using polyacrylamide (PA) to recapitulate different stiffnesses experienced during disease progression [102]. In both cell lines, Piezo1-regulated calcium flux was found to play a key role in prostate cancer cell metastatic potential by sensing changes in ECM stiffness and modulating EMT markers.

Cellular cocultures in microfluidic platforms, compared to microarrays, offer control over many more experimental variables. An adapted microfluidic platform was further developed to investigate the role of mechanical stimuli on human prostatic fibroblasts [103]. While unstretched normal tissue-associated fibroblasts (NAFs) deposited and assembled fibronectin in a random mesh-like arrangement, stretched NAFs produced a more organized, linearly aligned matrix. Stretching NAFs triggered complex biochemical signaling, e.g., increasing the expression of platelet-derived growth factor receptor a (PDGFRa), with enhanced capability for directing cancer cell migration. The observed phenotypes of stretched NAFs were like those associated with CAFs, suggesting mechanical stress to be a critical factor in NAF activation and CAF genesis. Droplet microfluidics is another promising tool recently adapted to model invasion of cancer cells from a tumor, through the BM, and into its stromal tissue [104]. Cancer cells were encapsulated in beads of a primary hydrogel-mimicking BM (Matrigel), which were then embedded in a secondary hydrogel-mimicking stromal ECM (collagen I). Highly invasive cells penetrated the surrounding matrix as single cells, whereas poorly invasive cells did that collectively. The model reasonably resembled an in vivo heterogeneous tumor microenvironment, allowing control over several parameters, such as the tissue size, cell count and type, as well as ECM composition and properties. Still, most in vitro models for invasion have failed to account for the smooth muscle found around organs such as the prostate, in which cancer progression from low- to high-risk is characterized by tumor invasion into the smooth muscle barrier [105]. Various physiologically relevant factors in the tumor microenvironment contribute to the ability of cohesive clusters of tumor cells to penetrate and move through the smooth muscle. Hence, understanding the tumor phenotype plasticity, with the significance of involved pro-inflammatory cytokines, would require proper model systems. Elucidating integrated mechanosensing features within the tumor cluster and the smooth muscle will likely yield new relevant factors required for smooth muscle invasion and, subsequently, offer new diagnostic tools to block metastasis.

#### 3.2.2. Intravasation

Intravasation is the process whereby cancer cells leave the primary tumor site and transverse the endothelium, marking the onset of the metastatic cascade. In the process, cancer cells reorganize and migrate through the interstitial ECM matrix, break through the basement membrane, and enter blood vessels or lymphatic capillaries. The identification of the mechanisms that trigger the entry of cancer cells into the bloodstream may reveal new strategies to block metastasis at its start. The role of cancer cells’ intrinsic properties, tumor microenvironment, and mechanical cues in the intravasation process have been summarized [106]. Once capable of migrating across the ECM, cancer cells were found to move along collagen fibers, leading from the primary site to nearby lymph or blood vessels. Various signaling pathways and cellular interactions contribute to the formation of a local microenvironment, which enables cancer cells to overcome tight intercellular junctions and penetrate blood vessels (Figure 2A); cells that can enhance tumor cell intravasation include macrophages, fibroblasts, neutrophils, and platelets. Cell surface proteomic analysis provides a powerful tool for identifying specific membrane molecules that functionally contribute to intravasation and, based on current assays, several pathways and molecules have been identified as important for intravasation [107,108]. Overexpression of osteopontin in prostate cancer cells (LNCaP and PC3) was observed to increase proliferation, invasion, and intravasation [109]. A comparison of PC3 intravasation variants pointed to key roles for the uPA-plasmin system, possibly via promoting tumor cell matrix invasion and facilitating the development of VEGF-dependent angiogenic blood vessels [110]; thus, inhibition of pro-uPA activation, at the apex of the uPA/plasmin cascade, could be an approach to control the onset of tumor escape and metastatic spread [111]. TGF-β signaling and EGFR activation were implicated in inducing invadopodia, actin-rich protrusions of the plasma membrane, reported to enhance intravasation through gradual ECM breakdown and the release of ligands for integrin receptors using focalized proteolysis [112,113]. Invadopodia formation is a dynamic process requiring the combined and synergistic activity of ECM-modifying proteins with cellular receptors and the interplay with factors from the TME. Biochemical and biophysical interactions exist not only between the invadopodia actin cores and specific intracellular structures, e.g., the cell nucleus, but also with elements of the TME, e.g., stromal cells. Particularly, direct contact between macrophages and cancer cells activates pathways, resulting in increased invadopodium formation in the tumor cells at blood vessels, promoting their sensitization to growth factor signals and tumor cell intravasation across an endothelial barrier [114].

Microfluidic technology is now available to apply sophisticated assays for the analysis of intravasation, enabling a more detailed understanding of the events taking place, as it is likely that different tumor types (or subtypes) utilize different mechanisms of intravasation. The tumor–vascular interface was established in a microfluidic system developed to enable quantification of the endothelial barrier function. Measurements of permeability indicated that macrophages’ signaling, mediated by tumor necrosis factor-α secretion, resulted in degrading the endothelial barrier [115]. Indeed, macrophages are known as key promoters of tumor metastasis, consistent with clinical data revealing that infiltration of macrophages in tumor tissues correlates with poor prognosis in several cancers including prostate. Using a microfluidic 3D cell migration assay, the presence of macrophages was found to enhance the speed and persistence of PC3 cell migration in a matrix metalloproteinases (MMP)-dependent fashion [116]. Mechanistic investigations further revealed that macrophage-released TNFα and TGFβ1 mediated the observed effect by two distinct pathways, suggesting that their dual blockade could be an anti-metastatic strategy in solid tumors. To maintain appropriate spatial gradients of growth factors, microvessels were generated in a metastasis chip where endothelial and stromal cells were patterned near tumor cells. Cancer angiogenesis and intravasation, as well as their modulation by treatment, were successfully reproduced, allowing quantification of the angiogenic response, as well as tumor cell trans-endothelial migration [117]. Coupling a 3D cylindrical design with confocal microscopy, tumor cells significantly increased the proangiogenic gene expression when co-cultured with endothelial cells under a controlled flow, highlighting the effect of fluid forces on tumor–endothelial crosstalk [118]. Some microfluidic platforms have been developed to study both intravasation and extravasation on a single device. Both assays were integrated in a cell-based microfluidic chip consisting of two parts: an intravasation chamber for the 3D cancel cell culture, using a Matrigel matrix, and an extravasation chamber for the detection of metastasized cancer cells by epithelial cell adhesion molecules [119]. Under physiological shear stress, cancer cells from the intravasation were found in the extravasation chamber, with differences in binding efficiency detected when using a cell-adhesion inhibitor for treating degraded cancer cells. A microfluidic device consisting of independent cell collection chambers under an embedded endothelium was designed to selectively collect only trans-endothelial migrating cells, allowing the characterization of features such as morphology, cytoskeletal structures, as well as migration speed and persistence [120]. The presence of a spontaneously formed vasculature was observed to enhance cell invasion into the 3D stroma in a 3D microfluidic system consisting of three-layer hydrogels laden with cells [121]. The vessel diameter was reduced significantly by the invading cancer cells, and signaling cytokines linked to tumor–vascular interactions controlling invasion and intravasation were detected. The presence of endothelial cells with fluid flow was reported to significantly affect tumor cell proliferation, migration, and intravasation in a microfluidic tumor-vessel co-culture system established for interrogating the different phases of cancer metastasis. Interestingly, some cancer cells (breast) were reported to invade through the paracellular route, disrupting intercellular endothelial junctions, while others (liver) were observed to engage in transcellular intravasation [122]. Prostate cancer cells migration in confined spaces without chemotaxis was investigated in a 3D glass biochip consisting of embedded microfluidic channels and pillar-like structures, separated by narrow channels, to recreate a 3D intravasation–extravasation configuration [123]. Prostate cancer cells (PC3) were observed to not only breach constructed submicron barriers, while maintaining their viability and proliferation capacity, but also penetrate the extravasation-like confining spaces. The results confirmed the dynamic adaptability of cancer cells, experiencing dramatic compression and stretching levels, as they pass through submicron constrictions with volumes smaller than a cell nucleus.

#### 3.2.3. Circulation

In metastatic disease, cancer cells enter the vascular or lymphatic system and are carried around the body through blood circulation. These cells are known as circulating tumor cells (CTCs), which can infiltrate distant organs to form secondary tumors. CTCs are presumed to be derived from both the primary and metastatic lesions and, if the primary tumor has been resected, only from the metastatic lesions; metachronous metastases, developing after completion of an initial treatment, are common in prostate, breast, colon, and skin cancer [124]. Analysis of independent metastatic sites within a single patient revealed that each site can evolve independently, acquiring different mutations [125]. Hence, primary tumor resections and single-site biopsies of metastatic lesions may not capture the overall mutations across multiple sites and, perhaps, CTCs could provide a clearer picture of the entire population of invasive cancer cells. In light of metastasis as the leading cause of cancer-related death, the analysis of CTCs can assist in the diagnosis of patients and adjust treatment tailored to the evolving cancer. Tumor cells’ intravasation into the circulation can take place via entry of lymphatic or vascular vessels depending on several factors such as accessibility to blood vessels [126]. In lymphatic intravasation, lymph vessels eventually drain into the blood allowing tumor cells to indirectly gain access to the venous blood supply; along the way, tumor cells encounter a series of lymph nodes closest to the primary tumor and often the first sites of metastasis [108]. CTCs are present in blood as individual cells, clusters of CTCs, and clusters composed of CTCs, leukocytes, platelets, and perhaps other cells known as circulating tumor microemboli (CTM) [127]. Despite observations suggesting that some 10^6^ tumor cells enter circulation every day, CTCs are rare in cancer patients blood; about 85% disappear within five minutes, and only one CTC can be detected among 10^5^–10^7^ mononuclear cells per ml blood [128,129,130,131,132]. Individual CTCs, which appear at double the rate of CTC clusters, face three major obstacles while suspended in blood: anoikis (a programmed cell death upon cell detachment from correct ECM), immune attack, and hydrodynamic shear stress [133,134,135]. CTMs are perhaps better protected from these destructive mechanisms and consequently have a greater capacity for metastasis than individual CTCs, as demonstrated in experimental models [136]. The intravascular microenvironment that CTCs must endure in the circulatory system has been analyzed, and various aspects of their intravascular transport have been detailed using a mathematical model based on immersed boundary principles [137]. Rather than fragile, CTCs are mechanically robust under exposure to physiologic shear stresses encountered during their passage through the circulation, and emerging evidence suggests that malignant epithelial cells possess specific mechanisms enabling them to endure these mechanical stresses [138]. Using a CTC-Chip, a quantitative automated imaging system for analysis of prostate CTCs was presented, taking advantage of prostate-specific antigen (PSA) [139]. The half-life of CTCs in the blood circulation after surgical resection of the primary tumor was estimated to be less than 24 h, while dual staining of the captured CTCs for PSA and a cell division marker (Ki67) indicated a broad range for the proportion of proliferating cells among them. A geometrically enhanced differential immunocapture (GEDI) microfluidic device was developed to characterize CTCs captured from castrate-resistant prostate cancer (CRPC) patients [140]. In addition to CTC enumeration, specific androgen receptor point mutation (T868A) was detected by sequencing, TMPRSS2-ERG fusion was identified by immunostaining, and ex vivo CTC sensitivity to drugs was assessed using microtubule bundling as a marker. The integration of sample preparation, image analysis, and molecular characterization of CTCs was accomplished in a microfluidic platform developed for analyzing CTCs collected from blood samples of metastatic patients [141]. The chip was processed using an automated staining and scanning system facilitating rapid capture of CTCs along with their downstream characterization by immunohistochemistry, DNA, and mRNA fluorescence in-situ hybridization (FISH).

Little is known about CTC locomotion following a period of arrest in the microvasculature. Due to the size limit of the microcirculation, CTCs about 10–20 μm in size must deform to pass through some blood vessels smaller than 5 μm in diameter. The mechanical response of living cells to an external force is generally loading-rate dependent, and a homogeneous linear viscoelastic model is popularly used to describe cell deformation due to mechanical loading [142]. The deformation of prostate cancer cells (PC3N) captured in an antibody-functionalized microchannel was dramatic under low acceleration and negligible under high-flow acceleration; a loading rate time constant of about 1 min was estimated for the cells to exhibit either viscous or elastic response [62]. Although the microvasculature poses an even greater obstacle to CTMs, due to their larger size, over 90% of CTC clusters containing up to 20 cells were able to pass through 5–10 μm constrictions in microfluidic devices designed to mimic the human capillary network; the clusters reorganized reversibly and rapidly into single-file geometries, substantially reducing their hydrodynamic resistance [143]. Various model systems have been utilized to assess the biological effects of fluid flow on CTCs since they are exposed to greatly varying levels of fluid shear stress (FSS) in the bloodstream [138]. Using a micropipette aspiration technique, prostate cancer cells were found to adapt to FSS with an increased Young’s modulus as a function of the FSS magnitude [144]. This adaptive biophysical property of CTCs in response to FSS is likely related to their ability to avoid damage from subsequent FSS pulses. There seems to be certain mechanisms in cancer cells that confer resistance to fluid shear stress, not present in benign cells, which appear to be the product of cellular transformation associated with oncogenic pathways common to many cancers including prostate.

#### 3.2.4. Extravasation

In the past decade, since pre-metastatic niche (PMN) was first described, it has been established that metastatic organs are actively modified by the primary tumor even before metastatic spread has taken place. Tumor-secreted factors and extracellular vesicles (EVs) induce PMN formation in target organs before CTC extravasation occurs. CTCs seeding the PMN survive and give rise to micrometastasis, usually defined as a cluster of 10–12 cells, while CTCs seeding non-PMN areas lack an accommodating microenvironment and fail organ colonization. A model of PMN formation has been proposed, where its progression is initiated with local changes such as the induction of vascular leakiness, remodeling of the stroma and ECM, followed by dramatic systemic effects on the immune system [145]. Experiments suggest that matrix metalloproteinases (MMPs) expressed by tissue-resident cells at sites of PMN formation act in concert to destabilize the vasculature and create a PMN. The PMN formation and evolution seems to present a new paradigm for the initiation of metastasis, underscoring the need to better understand the pathological processes occurring before the development of macrometastases, the outgrowth of micrometastases that are histologically or radiologically detectable. Viable CTCs travel via circulation to distant sites, extravasating the vasculature through the wall of blood vessels into the surrounding tissue to form secondary tumors at remote organs (Figure 2B); they transmigrate the endothelium in a process, mirroring the stepwise leukocytes extravasation. It is initialized by tethering and rolling due to relatively low-affinity binding of CTCs mediated by selectins. Rolling is then followed by tight adhesion through integrins and, after adhesion, CTCs transmigrate through the vascular endothelium by a procedure termed ‘diapedesis’ [146]. CTCs interact with platelets within minutes of their dissemination and have the capacity to directly hijack immune cells to form aggregates, which are more resilient with higher metastatic potential [147]. Blood platelets stimulate the extravasation of cells with depleted PRH, which inhibits migration/invasion, in part, by activating the transcription of Endoglin and, in prostate cells, transforming growth factor β (TGFβ) signaling downregulates PRH activity by multiple mechanisms, inducing EMT that facilitates extravasation [148]. CTCs arrest at secondary sites occur primarily in selective organs, with prostate secondary sites appearing mainly in the bones [149]. Attachment of CTCs to the endothelium is mediated by several cell-to-cell adhesion molecules (CAMs) and their ligands organized in a sequential row, the CTC adhesion cascade, in which selectins and integrins with their ligands take center stage, governing the transmigration. Selectins, a three-member family of transmembrane glycoproteins (endothelial-, platelet-, and lymphocyte-selectins), have been directly implicated in extravasation for supporting tumor cell rolling on activated endothelium. The degree of selectin ligand expression by cancer cells is well correlated with cancer metastasis and poor prognosis [150]. CTCs move to the margin of the bloodstream, margination, where tethering and rolling are initiated by selectins expressed by CTCs and endothelial cells. By interacting with glycoproteins and glycolipids, selectins are responsible for the slow tethering and rolling of leukocytes on the vascular endothelium, enabling their extravasation during inflammation. Cancer cells take advantage of this physiological process to spread and colonize distant organs, and the function of selectins and their ligands in CTC extravasation is well documented [151]. Beyond the extravasation process, selectins/selectin ligands interactions have also been implicated in forming a permissive microenvironment for metastasis and retention of tumor cells in protective niches. During the selectin-mediated rolling, integrins are activated binding to their endothelial ligands to facilitate the tight adhesion and transmigration of CTCs. Integrin expression patterns found on primary tumor cells, and their correlation with tumor progression, have been reviewed [146]. Integrins are a large family of transmembrane cell adhesion proteins and either tie the matrix to the cytoskeleton or serve as cell-to-cell adhesion molecules, consisting of two non-covalently associated α and β; the α subunit determines integrin–ligand specificity, and the β subunit is connected to the cytoskeleton, affecting different signaling pathways. Although leukocytes express specific integrins for the interaction with ligands during extravasation, these leukocyte–integrins could be substituted on CTCs by other integrins or adhesion molecules with the ability to bind to the same ligands on the vascular endothelium. While it appears that the binding pattern of leukocytes and CTCs could be identical, tumor cells possess alternative ways of extravasation such as the use of leukocytes/platelets as linkers. In prostate cancer, bradykinin has been shown to enhance migration and spread by augmenting ICAM-1 expression, and micrometastasis could be inhibited by blocking integrin αVβ3 and ICAM-1. Leukocytes transmigrate through the endothelium, without irreversibly impairing its integrity, following a paracellular or transcellular route. In contrast, tumor cells presumably do not leave the endothelium intact after diapedesis [152]; it is widely assumed that the endothelium is irreversibly damaged, probably due to the induction of apoptosis by the loss of cell-cell contact during the transmigration of CTCs [153]. Emerging evidence suggests that invadopodia are essential not only for intravasation but also for extravasation into specific microenvironments permissive for metastatic colony growth. In visualizations of extravasation dynamics in vivo, cancer cells were observed to form protrusive structures by enrichment of certain cytoskeletal and scaffolding proteins, which exclusively localized to invadopodia, and to extend the invadopodia through the endothelium into the extravascular stroma prior to their extravasation at endothelial junctions [154]. Inhibition of invadopodia resulted in the abrogation of cancer cell extravasation and metastatic colony formation in an experimental mouse model, providing direct evidence of a functional role for invadopodia during cancer cell extravasation and metastasis. While prostate cancer cell lines have not been reported to consistently generate invadopodia without external stimulation, in a recent study, all prostate cancer cell lines tested were capable of spontaneous invadopodia formation, possessing a significant degradative ability in vitro under basal conditions [155]. Furthermore, CTCs isolated from prostate cancer patients exhibited invadopodia-like structures degrading the matrix with visible puncta and, thus, invadopodia activity could be one of the dissemination mechanisms employed by prostate cancer cells.

Cancer cell extravasation in response to biophysical and biochemical cues has been studied using microfluidics, and the characteristics of some devices, along with the discoveries enabled by them, have been discussed [156]. The effect of confinement on metastatic cancer cells was explored utilizing a cell motility assay developed for measuring the migration speed of individual cancer cells inside microchannels with cross-section comparable to cell size [157]. Among prostate cancer cells, the motility was the highest for PC3 and lower for LnCaP cells. The observed persistent and spontaneous motility suggested the presence of intrinsic mechanisms driving cancer cell migration induced by physical confinement. In addition to the biomaterial surface stiffness encountered by CTCs, cell stiffness and deformability have also been proposed as factors regulating cell migration [158,159]. Cell migration in microfluidic devices was impeded in cell lines with larger nuclei, lower adhesiveness, and to a lesser degree, also in cells with lower contractility and higher stiffness; hence, the ability to overcome the matrix steric hindrance was found to depend on a combination of factors, including adhesiveness, nuclear volume, contractility, and cell stiffness. Fluid flow is another factor playing an important role in cell migration, and a microfluidic system was established to apply physiological shear stress on tumor cells, demonstrating that an arterial level of shear stress enhanced significantly tumor cell extravasation in a trans-endothelial assay [160]. Intracellular levels of reactive oxygen species (ROS) were elevated by circulatory treatment, activating the extracellular signal-regulated kinases (ERK1/2); the ERK1/2 activation enhanced tumor cell migration and extravasation in a trans-endothelial assay as well as in a zebrafish model. As it is indispensable to incorporate endothelial cells in blood vessel models since they present the first barrier encountered by extravasating CTCs, the principal components of biological blood vessels, including vessel cavity, endothelium, and perivascular matrix containing chemokines were recapitulated in a microfluidic model allowing real-time recording of transendothelial migration [161]. Tumor aggregates transmigrated across the endothelium under the stimulation of chemokine CXCL12, irreversibly damaging the endothelial integrity at the crossing site, while the transmigration was inhibited by blocking its CXCR4 receptor with AMD3100, highlighting the importance of the CXCL12-CXCR4 axis in cancer metastasis. Isolation of primary CTCs from buffy coat samples of metastatic cancer patients was accomplished in a bio-functionalized microtube, allowing the replication of CTCs adherence to the endothelium, the pre-cursor step for trans-endothelium migration [162]. The presence of E-selectin-mediated interactions in prostate CTCs under physiologic shear stress conditions was confirmed, providing a route to initiate trans-endothelial migration and metastasis [163]. Moreover, the ex vivo interactions between CTCs isolated from patients and E-selectin on activated endothelial cells correlated with the clinical response of castration resistant prostate cancer patients, suggesting a dynamic modulation of selectin ligands expression as the disease develops to a more aggressive phenotype. In vitro exploration of organ-specific extravasation of CTCs was conducted in a microfluidic system featuring local chemokine gradients in a duct coated with endothelial cells [164]. The system was used to illustrate paracellular trans-endothelial migration of individual cancer cell expressing CXCR4, without degrading the endothelium, in response to CXCL12 chemokine gradients. A different microfluidic strategy was developed to simultaneously analyze CTC−endothelial adhesion, endothelial trans-migration, and motility through a 3D ECM under gradients of the chemoattractant SDF-1α [165]. Once cancer cells encountered the activated endothelium, subsequent extravasation was observed presumably guided, in part, by the SDF-1a permeability through the endothelial monolayer or due to the secretion of secondary cytokines by activated endothelial cells. The consequences of integrin β1 depletion in cancer cells were assessed in a 3D microfluidic model of the human microvasculature engineered to recapitulate the environment wherein extravasation takes place [166]; β1 was required for invasion through the basement membrane, whereas its suppression in a syngeneic tumor model resulted in reduced colonization, revealing a critical role of β1 in several steps of extravasation. Several key metastatic steps of tumor cells in blood vessels, including extravasation, were reconstituted in a microfluidic vessel-on-a-chip platform [167]. Prostate cancer single or cluster cells (PC3) extravasated EC-coated microchannels under HGF stimulation. Individual tumor cells extravasated without disrupting the endothelium integrity, whereas the extravasation of tumor clusters was harsh, compromising the vessels’ integrity. Furthermore, both vascular glycocalyx (VGCX) shedding and vessel shape-induced flow disturbances were reported to enhance CTC homing to local vascular tissue.

##### Bone Tropism

Metastasis occurs not only in the skeleton; however, in prostate and breast cancer, it is the most common site for metastasis, with 65–75% relative incidence in bone [168]. Disseminated tumor cells (DTCs) extravasating in the bone settle in a microenvironment that is mechanically different from the primary tumor microenvironment that is controlled by bone cells [169]. Metastasis to bones occurs when the normal balance between bone-generating osteoblasts (OB) and bone-degrading osteoclasts (OC) is modified; while breast and prostate tumors share the commonality of steroid dependence and bone affinity, their interaction with the bone TME is distinctly different [170]. In breast cancer, osteolytic metastasis occurs as tumors remodel the bone by stimulating the production of OCs whereas, in prostate cancer, osteoblastic metastasis occurs as tumors remodel the bone by stimulating the production of OBs, leading, in either case, to bone fractures and pain. A limitation in studying cancer–bone metastasis is the multifaceted nature of the in vivo bone microenvironment and, consequently, the lack of in vitro models faithfully mimicking the biological processes, including mechanical loading. Three-dimensional in vitro models, particularly ex vivo patient-derived models, have emerged as superior systems to reconstitute the complexity of inter- and intra-patient heterogeneity of the bone metastatic niche [171,172]. The selection of specific organs by metastatic cancer cells is closely dependent on chemokines and their receptors. Human bone marrow endothelial cells and differentiated osteoblasts were found to express fractalkine, whereas human prostate cancer cells overexpressed its specific receptor CX3CR1 [173]. Androgens were investigated to dissect the mechanisms regulating the fractalkine level in bone marrow, where dihydrotestosterone (DHT) was found to dramatically increase the cleavage of fractalkine from the plasma membrane of bone cells, with its action reversed by an antagonist of the androgen receptor (nilutamide) [174]. Thus, androgens could promote the extravasation of CX3CR1-expressing cancer cells along a fractalkine concentration gradient, indicating a potential role in prostate cancer bone metastasis. Prostate tumor cells initiate interactions with bone marrow endothelium under blood flow through binding with E-selectin, and the E-selectin-binding form of P-selectin glycoprotein ligand-1 (PSGL-1) was reportedly expressed on human bone-metastatic prostate tumor cells implicating it in bone tropism [175]. Using histology and quantitative microCT analysis, α_v_β_3_ integrin was also implicated not only in tumor growth within the bone but also in tumor-induced bone gain, a response resembling bone lesions in prostate cancer patients [176]. Exerting pressure on osteocytes in a mouse model induced prostate cancer growth and invasion, and mechanistic analysis further revealed that the process was mediated by the upregulation of CCL5 and matrix metalloproteinases in the cells [177].

The need for in vitro models mimicking the in vivo tumor niche has become apparent with the ever-growing evidence of the prominent role in cancer growth played by the niche microenvironment in which cancer cells reside. An early metastatic prostate cancer model was engineered featuring a two-layer microfluidic system designed for culturing spheroids of prostate cancer cells (PC3), osteoblasts, and endothelial cells; the proliferation rate of PC3 cells matched the in vivo growth behavior of malignant prostate cancer cells within the bone microenvironment [178]. To investigate the mechanisms by which prostate cancer cells metastasize to bone, an extracellular matrix (ECM) hydrogel with a continuous lumen was formed by flowing fluid through the hydrogel in a microfluidic channel; then, seeding cancer cells into the lumen made it possible to quantitatively analyze their invasion into the ECM hydrogel [179]. The unique mechanical properties of bone ECM, mainly composed of hydroxyapatite (HA), affected several cellular responses in the TME directly related to bone metastasis. Interactions between the TME and HA were investigated utilizing a bone-mimetic microenvironment established in a microfluidic platform designed for culturing tumor cells in a 3D HA and fibrin composite [180]. The HA-rich microenvironment was observed to influence cell viability, proliferation, and cytoplasmic volume in a manner dependent on the cancer cell type and culture duration. At higher HA concentration, cancer cells exhibited significantly reduced migration and angiogenic sprouting, indicating an inhibitory effect of HA. The osteocytes’ role in mechanical regulation of bone metastasis was investigated in an in vitro microfluidic tissue model, where metastatic tumor cells were cultured in a 3D lumen lined with HUVECs next to a channel seeded with osteocyte-like cells [181]. Oscillatory fluid flow (OFF) was applied to mechanically stimulate the osteocytes, clearly inducing intracellular calcium responses. Under the applied OFF, the extravasation distance and the fraction of extravasated side channels were reduced, significantly demonstrating that the mechanical stimulation of osteocytes may reduce cancer cell extravasation. Interstitial fluid flow could also affect cancer metastasis, and a horizontal flow bioreactor was designed to elucidate its impact on prostate cancer cells migration to the bone during extravasation [182]. The high flow rate induced apoptosis in prostate cancer cells (PC3) via TGF-β1-mediated signaling without affecting CXCR4 activation. However, in the presence of the bone, the CXCR4 expression level was upregulated, leading to increased MMP-9 levels and resulting in a high migration rate. In addition, upregulated levels of α_v_β_3_ integrins further enhanced the migration rate of PC3 cells, suggesting that interstitial flow-induced shear stress could be important in regulating prostate cancer cells migration during extravasation. As microfluidic systems are still restricted in creating tissues of limited complexity, a macrofluidic model of skeletal metastasis was developed to study cancer cell migration from a primary tumor to remote sites using tissues [183]. Excised tumor xenografts from mice or prostate carcer cells in a hydrogel were placed in the primary tumor sites, while human-derived bone chips or bone stromal cells (HS-5) in a hydrogel were seeded in the metastatic sites. Cells from primary migrated to metastatic sites, while the bone enhanced cell growth at metastatic sites, establishing a higher CXCL12 gradient in metastatic sites compared to primary sites. Inhibition of the CXCL12 function by AMD3100 reduced the number of cells targeting the bone metastatic sites. As complimentary to microfluidic models, enabling high-throughput sophisticated experiments, this model system enables evaluation of metastasis using solid tumors and cell suspensions in an ex vivo microenvironment with tissues of both primary and secondary sites.

#### 3.2.5. Dormancy and Colonization

**Figure 3:**
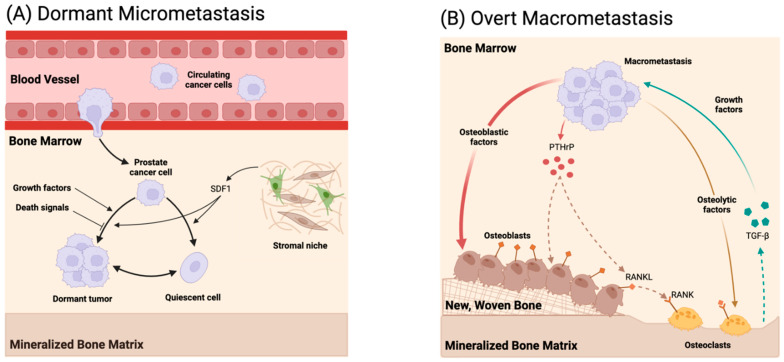
Survival and emergence of prostate DTCs in the bone: (**A**) In indolent micrometastasis, once CTCs have settled in the bone niche, they may be exposed to a balance between growth and death signals in the new stromal microenvironment, preventing metastatic colonization and forcing the DTCs into either a growth arrest or an indolent micrometastatic growth state; cells bearing proper genetic or epigenetic composition can utilize other cues for survival, e.g., DTCs expressing CXCR4 could interact with the pro-survival chemokine CXCL12 that is produced by mesenchymal cells resident in the bone marrow. (**B**) In overt macrometastasis, the emergence from latency is due to the continuous evolution of DTCs into metastatic cells that have gained the capacity for colonization over an extended time period. In prostate cancer, tumor cells release osteoblastic factors (such as PTHrP), activating osteoblasts to form new bone (osteoblastic lesions); the stimulated osteoblasts also upregulate RANKL expression, which interacts with RANK receptor on osteoclast progenitors and, together with released osteolytic factors, promote bone resorption through osteoclastic differentiation (osteolytic lesions). In turn, activated osteoclasts release cytokines that are normally stored in the bone matrix, such as TGF-β, to further promote tumor growth perpetuating a ‘vicious cycle’ of macrometastasis outgrowth.

A notable metastatic feature is the ability of various tumor types to colonize different or the same secondary sites, while some tumors have a limited range of target sites; for instance, prostate cancer metastasizes mainly to the bone. The ability of cancer cells to infiltrate certain organs is not always accompanied by the ability to colonize these organs, and the time elapsed between organ infiltration and colonization likely indicates a period of metastatic dormancy, as illustrated in Figure 3 [149]. Cancer dormancy is a stage in tumor progression in which residual disease remains undetected and asymptomatic for a prolonged period (Figure 3A). Quiescence and senescence are distinct cellular dormant states; senescence is the irreversible gradual deterioration of cellular functions, ensuing cell death due to aging or serious DNA damages, while quiescence is reversible under proper growth signals [184]. Quiescence is a cell state, actively maintained, heterogeneous, and characterized by a graded depth; as cells remain quiescent for longer periods of time, they sink progressively deeper with reduced sensitivity to growth signals [185]. In hormone-driven cancers, such as prostate and breast, metastasis might manifest decades after the removal of even a small primary tumor. In these malignancies, the acquisition of competency for colonization is likely to occur during the residence of DTCs in a particular organ microenvironment involving the acquisition of specific functions (Figure 3B) [149]. Although the mechanisms regulating the transition of DTCs from dormant into a proliferative state remain largely unknown, the regulation of dormant tumor cells growth may be explained, in part, through the interaction of tumor cells with their microenvironment, limitations in blood supply, or an active immune system [5]. Freshly disseminated cancer cells are particularly vulnerable to the innate immune system, which can control and shape cancer through: (i) elimination, in which immunity functions as a tumor suppressor; (ii) equilibrium, where the expansion of tumor cells is held in check by immunity; and (iii) escape, in which tumor cells with the capacity to attenuate immune responses grow into overt metastasis [186]. Immunologic equilibrium postulates that tumor cells proliferation is suppressed by immune surveillance, entering a dormant state via procurement of a reversible growth arrest, as well as attenuation of their immunogenic potential. The equilibrium will be sustained until new tumor cell mutations and/or additional immune suppression trigger immune escape and initiate metastatic disease. Different types of immune cells are involved in molecular crosstalk with cancer cells at every stage of the metastatic cascade; some induce dormancy, and some facilitate their emergence from a dormant state [187]. Tumor dormancy can be classified in two main modes: (i) cellular dormancy, in which isolated DTCs enter a quiescent state, and (ii) tumor-mass dormancy, where a balance between cell proliferation and death is maintained such that micrometastases cease to grow; it is not clear which mode leads to overt metastases most frequently. Organs significantly differ in the structure and composition of their tissues, and their colonization involves definite organ-specific metastatic properties [188]. Cancer stem cells (CSCs) constitute a subpopulation of tumor cells with the capacity of self-renewal and multi-lineage differentiation, as well as resistance to cytotoxic compounds; they are defined by their tumor-propagating ability and by their ability to recapitulate the cell-type heterogeneity observed in the original primary tumors [189]. After cancer stem cells land in remote sites, their survival and tumorigenesis potential can be aided by interactions with specialized niches, and results indicate that prostate-carcinoma stem cells settle in native hematopoietic stem cell niches in the bone marrow [6]. The CSC quiescence state is primarily achieved through molding the tumor microenvironment and manipulating its underlying regulatory pathways; quiescent CSCs can survive harsh conditions and, subsequently, repopulate the tumor mass, resulting in tumor recurrence [190].

The onset of metastatic organ colonization, in which the TME plays a critical role, has been recognized as the event defining cancer progression into a lethal disease, and the TME complexity has, for a long time, limited the development of in vitro models for studying cancer dormancy and relapse. Major advances in tissue engineering within the last decade have enabled the realization of bioengineered models, in which key functions of the TME can be reconstituted. These models have been classified according to either the dormancy-induction mode (i.e., ECM, cell signaling, biochemical, and drug) [191] or the tissue-engineering strategy (i.e., 3D biomaterial, bioreactor, and microfluidics) [192]. Three-dimensional cell co-cultures in microfluidic systems have enabled the detection, characterization, and manipulation of dormant cancer cells in wide range of applications [193]. Organs-on-chips allow tumor dormancy research at a gradually increasing level of complexity, from cells to organs, offering real-time and high-resolution analysis while incorporating relevant biochemical and biophysical cues, and microfluidic devices have been constructed to interrogate the response of multipotent stem cells to signals, regulating their fate whether stay quiescent, differentiate, or undergo self-renewal [194]. Cancer cells entry into and emergence from dormancy in the liver was explored using a commercially available ex vivo hepatic MPS (LiverChip, CN Bio Innovations Ltd., Cambridge, UK), which was later utilized with breast and stellate cell co-culture to identify factors mediating tumor escape from dormancy [195,196]. Activated, but not quiescent, hepatic stellate cells were found to highly secret soluble factors (IL-8 and MCP-1) inducing the proliferation of otherwise dormant cancer cells, and IL-8 significantly increased cancer proliferation ex vivo, suggesting that inflammatory cytokines promote tumor escape from hepatocyte-induced dormancy. Upon seeding the hepatic MPS with breast cancer cells, actively proliferating and spontaneously quiescent populations were formed [197]. Following chemotherapeutic treatment, the proliferating cells were eliminated, but the quiescent cells remained; the residual dormant population could then be induced to a proliferative state by physiologically relevant inflammatory stimuli (LPS and EGF). Applying mechanical actuation in a human model of non-small-cell lung cancer (NSCLC) revealed that cancer cell responses were affected by physical cues associated with breathing motions, mediated by changes in signaling through epidermal growth factor receptor (EGFR) and MET protein kinase [198]. Droplet microfluidic technology was developed to investigate metabolic differences between quiescent and proliferating cells considered to be important for understanding single-cancer-cell dormancy in a heterogeneous culture [199]. A microfluidic approach coupled with phenotypic classification by machine learning was employed to identify stress pathways associated with starvation-triggered quiescent states [200]. Both low- and high-Cdk1 quiescent states were found to share a core of stress-associated processes, e.g., autophagy, but differed in the nuclear accumulation of stress transcription factors such as Xbp1. The choice between low- or high-Cdk1 quiescence was determined by the cell cycle-independent accumulation of Xbp1, which acted as an integrator of the stress–stimuli duration, demonstrating how stress-activated factors promote cellular quiescence. Previously observed quiescence characteristics under static medium, e.g., serum signal-dependent quiescence entry and exit along with time-dependent quiescence deepening, were also found under steady medium flow in a microfluidic system developed to study the interstitial fluid effect on the depth of cellular quiescence [201]. The experimental results were simulated by a theoretical model relating the extracellular fluid flow effect to an Rb-E2f bistable switch, which regulates the transition from quiescence to proliferation, providing a partial explanation to the heterogeneous quiescent cell response in living tissues for regeneration and repair.

**Table 1:** Summary of selected microfluidic systems used in prostate cancer research.

Reference	Cells/Samples Used	Culture Type	Field of Investigation	Device Properties	Findings
Dolega [48]	Non-malignant RWPE-1, PC3, MCF-10A	3D	Prostate cancer physiology	Glass capillaries mounted with needles were used to form circular channels through PDMS. Devices were coated with a thin membrane of Matrigel or layered polyelectrolyte.	Cells adhered to and proliferate in channels of diameter ≥150 μm. Continuous flow through channels did not induce cellular detachment.
Jiang [49]	Immortalized PrEC, BHPrS1	3D	Prostate cancer physiology	Two stacked, z-shaped PDMS channels separated by a polyester membrane with a 0.8 μm pore size. Each channel consisted of 500 μm height, 1 mm width, and 40 mm length, with a 20 mm overlapping segment of the microchannels.	Established an in vitro model of the human prostate. Demonstrated R1881-mediated epithelium differentiation.
Picollet-D’hahan [59]	PC3, PNT-2	2D	Prostate cancer physiology	Polyelectrolyte films on multi-well plates were fabricated with anionic PSS (poly(sodium 4-styrenesulphonate))-terminated and cationic PAH (poly(allylamine hydrochloride))-terminated coatings in 2 nm-thick layers.	PAH-terminated surfaces had better initial attachment of PC3 but prevented clustering, reduced focal adhesion points, and decreased proliferation rate compared to PSS. No polyelectrolyte surfaces affected biological responses of PNT-2.
Liberio [60]	LNCaP	2D	Prostate cancer physiology	96-well plates were coated with one of the following substances: 1.3 μL laminin; 1 μL human placenta type IV collagen; 0.4 μL fibronectin; 50 μL poly-L-ornithine; or 0.32 μL poly-L-lysine.	Fibronectin, poly-L-lysine, and poly-L-ornithine improved LNCaP adherence and reduced cell mobility. Laminin and collagen type IV promoted cell aggregation. No coatings affected the expression of androgen receptor-regulated genes.
Cheung [62]	PC3N, MDA-MB-231-N	3D	Prostate cancer physiology	Straight channel (100 μm height, 32 mm length, 1 mm width) PDMS microchannel surfaces were immobilized with antibodies for cell capture.	Detachment of cells depended on flow rate and acceleration. A proposed exponential-like empirical model could predict the flow rate required for cell detachment as a function of flow acceleration.
Liu [63]	PC3	3D	Prostate cancer physiology	PDMS and glass device with 32 μm-thick PDMS diaphragm between upper and lower layers. The upper PDMS channel consisted of 4 rectangular chambers connected to external gas sources for generating shear forces.	The microfluidic chip could culture cancer cells for at least one month and output cancer cells externally using only hydrodynamic forces.
Liu [64]	LNCaP, DU145, PC3	2D	Prostate cancer physiology	Single-channel PDMS device (300 μm length, 25 μm height, 25 μm width) under a flow rate of 0.2 μL/s. Microfluidic methods were verified using atomic force microscopy.	Androgen-insensitive prostate cancer cell lines (PC3 and DU145) had a higher elastic modulus than androgen-sensitive (LNCaP) cell lines.
Dolega [65]	RWPE1, WPE1-NB26	3D	Prostate cancer physiology	3D cell cultures were grown in Matrigel and thawed into 4-well or 8-well Labtek plates.	Lensfree imaging allowed for the observation of epithelial tissue morphogenesis via the distinction between acini and spheroids in cancerous and normal models. Cell–cell and cell–environment interactions during differentiation could be studied by modulating stimuli within the ECM.
Tricoli [66]	Patient-derived normal and malignant prostate CRCs	3D	Prostate cancer physiology	2 polycarbonate cell culture inserts were placed in a 6-well plate and coated with 0.1% gelatin in 3 alternating applications.	Both normal and tumor-derived prostate epithelial cells underwent rapid basal-to-luminal differentiation (2 weeks) in 3D culture.
Fang [73]	C4-2 transfected with pcDNA3.1 or PKD1-GFP, WPMY-1	3D	Prostate cancer physiology	Alginate hydrogel in a microfluidic device with PLO perm selective basement membrane. Device specifications not mentioned.	Co-culture of epithelial and stromal cells allowed for the measurement of the shed component of E-cadherin. The amount of E-cadherin present in conditioned media was influenced by epithelial–stromal interaction.
Businaro [74]	B16 melanoma cells, immune cells isolated from spleen of wild-type (WT) and IRF-8 KO mice.	3D	Prostate cancer physiology	PDMS device with two culture chambers (1 mm width, 8 mm length, 100 μm height) and center channels (4 mm length, 1 mm width) connected via four sets of microchannels (12 μm width, 500 μm length, 10 μm height).	WT spleen cells more readily migrated toward B16 cells compared to IRF-8 KO immune cells.
Jeong [75]	HT-29 human colorectal carcinoma cells, CCD-18Co human normal fibroblasts	3D	Prostate cancer physiology	PDMS devices were loaded with hydrogel mixture and bonded to either a glass coverslip or a PDMS membrane. Channels were coated with poly-dopamine.	HT-29 grew into 3D tumor spheroids (TS) when co-cultured with fibroblasts. Fibroblasts demonstrated increased αSMA expression and migratory activity.
Ivich [77]	Immortalized PrECs, BHPrS1	3D	Prostate cancer physiology	Two stacked, z-shaped PDMS channels separated by a polyester membrane with a 0.8 μm pore size. Each channel consisted of 500 μm height, 1 mm width, and 40 mm length with a 20 mm overlapping segment of the microchannels.	Normal epithelium differentiation was induced and regulated by normal stroma. Normal stroma assumed a cancer-associated fibroblast phenotype when co-cultivated with cancerous epithelium.
Jiang [78]	Immortalized PrECs, BHPrS1, EMP, C4-2, 22Rv1	3D	Prostate cancer physiology; Invasion	Two stacked, z-shaped PDMS channels separated by a polyester membrane with either a 0.8 μm or 8 μm pore size. Each channel consists of 500 μm height, 1 mm width, and 34 mm length with a 20 mm overlapping segment of the microchannels. Tumor invasion studies were performed using devices with a serpentine channel overlaying a straight channel.	Tumor cells downregulated androgen receptor (AR) expression and induced CAF biomarkers in stromal fibroblasts. Both tumor cells and stromal CAFs migrated through the 8 μm pore size membrane and into their neighboring channel.
Graham [87]	ARCaP_M_ androgen-independent mesenchymal prostate carcinoma	3D	Invasion	Invasion assays performed using Boyden chambers with 8 μm pore size polycarbonate membrane inserts coated with Matrigel.	ZEB1 downregulation in ARCaP_M_ cells resulted in a significant (8-fold) reduction of invasion through Matrigel compared to control siRNA-treated cells.
Siddiqui [88]	PNT2-C2, PC3, DU145	3D	Invasion	Chemotaxis assays performed using 8 μm Boyden chambers in 24-well plates with Matrigel coating.	Overexpression of CK2 blocked the repression of migration and invasion by PRH. PRH knockdown in prostate cells resulted in increased invasion and decreased E-cadherin expression.
Wei [90]	1E8-H, 2B4-L human prostate epithelial cell lines	3D	Invasion	24-well Transwell chambers with 8 μm pore size polycarbonate filters. No mention of coating.	Vimentin transfection drastically increased invasion ability of 2B4-L cells and decreased invasion ability of 1E8-H cells.
Wang [91]	LNCaP, CWR22Rv1	3D	Invasion	12-well chambers with 12 μm pore size filters and Matrigel coating. LNCaP-SOX9 cell invasion was monitored for 24 h. Noninvaded cells were scraped off, while invaded cells were collected and quantified with fluorescence dye.	A greater number of LNCaP-SOX9 cells migrated through Matrigel-coated filters compared to uninduced and parental cells, indicating that SOX9 expression enhances tumor invasion.
Wang [92]	LNCaP, PC3, DU145, RPWE-1	3D	Invasion	24-well Transwell plate with 8 μm pore size polycarbonate filters. Upper chambers were coated with 50 μL Matrigel.	Knockdown of HOXA1 reduced the growth, invasion, and migration ability of DU145 and PC3.
Bonaccorsi [94]	PC3, DU145	3D	Invasion	Invasion assays performed using Boyden chambers overlayed with Matrigel-coated membranes.	The addition of an inhibitor of EGFR gefitinib decreased invasion of androgen-insensitive cell lines by inhibiting EGFR autotransphosphorylation and PI3K activation.
Hara [95]	MDA PCa 2b, androgen-independent MDA PCa 2b subline, 293T, LNCaP	3D	Invasion	Invasion assays performed using Matrigel invasion chambers with 8 μm pore size membranes.	Invasive (MDA-I) cells demonstrated increased AR expression compared with MDA PCa2b parental cells as well as maintaining AR dependence.
Li [96]	PshTertAR (immortalized, AR-positive), PshTert (immortalized, AK-negative), PC3	3D	Invasion	BD Biocoat Matrigel invasion chambers were used with stromal cells added to lower chamber and PC3 placed on the insert.	Stromal cells lacking AR increased the invasion ability of PC3 in vitro.
Palm [97]	PC3, PC3-*luc*	3D	Invasion	Norepinephrine and propranolol were mixed with PC3-*luc* in buffered type I collagen solution and filled into self-constructed chambers.	Treatment of cells with norepinephrine led to significant increase in migratory activity.
Ayala [98]	DU145, mouse ganglia/nerves, dorsal root ganglia/nerves (DRG)	3D	Invasion	Perineural invasion coculture model consists of DU145 and DRG embedded in Matrigel. DU145 cultured as monolayer adherent cells in microchamber system.	3 (NFκB, PIM-2, DAD-1) out of 15 genes overexpressed in DU145/DRG cocultures were known components of anti-apoptosis signaling pathways.
Sima [100]	PC3	3D	Invasion	The device consisted of 4 microreservoirs for cell culture and chemoattractant inputs connected by embedded glass channels. A panpipe-shaped polymeric scaffold in the device observation area consisted of 6 channels ranging from 6 to 21 μm in length.	PC3 migrated through channel lengths of 11 μm, 14 μm, and 21 μm in under 2 h by splitting into vesicular fragments and reassembling into single bodies.
Molter [101]	PC3, DU145, 22Rv1, LNCaP	2D	Invasion	PDMS substrates of varying stiffness were prepared by mixing different concentrations of Sylgard 184 PDMS cross-linking agent with the PDMS mixture. Stiffnesses of 1 kPa (resembling soft lymph node and brain tissue), 3 kPa (resembling normal prostate tissue), 12 kPa (resembling stiffened prostate tumor microenvironment), and 50 kPa (resembling non-mineralized bone environment) were used.	Substrate stiffness influenced the migratory behavior, contractility, and modulation of cell stiffness of prostate cancer cells. Aggressive cells (DU145 and PC3) exerted higher contractile stress compared to tumorigenic and lowly metastatic cells (22Rv1 and LNCaP).
Lopez-Cavestany [102]	DU145, PC3	2D	Invasion	Substrates of varying stiffness were used to investigate the role of stiffness in progression of the EMT phenotype. Two PA gels were used at stiffnesses of 5 kPa and 60 kPa, and a final glass substrate with a stiffness of 72 GPa was used as a positive control.	Increased matrix stiffness increased calcium steady-state concentration, increased vimentin expression, and decreased E-cadherin expression.
Ao [103]	NAFs, CAFs, SCC61	3D	Invasion	PDMS with vacuum chambers on either side of a suspended middle chamber (5 mm length, 1 mm width, 100 μm height) with a suspended PDMS membrane (10 μm thick)	Stretched normal tissue-associated fibroblasts altered the structure of secreted fibronectin and enhance cancer cell migration.
Jouybar [104]	MCF-7, MDA-MB-231, MCF-10A	3D	Invasion	PDMS devices were fabricated using soft lithography methods to achieve a channel height of 100μm, and the PDMS slabs were bonded to glass slides. A temperature control platform was created using a Low Force Stereolithography 3D printer and bonded to glass slides. Cells were encapsulated in Matrigel and placed in a droplet maker chip to create hydrogel droplets. Cells containing beads were sandwiched between two collagen I layers.	Highly invasive (MDA-MB-231) cells invaded the surrounding matrix in a single-cell manner, whereas poorly invasive (MCF-7) cells did so collectively.
Khodavirdi [109]	LNCaP (androgen-sensitive adenocarcinoma), PC3 (human prostate carcinoma)	3D	Intravasation	Matrigel invasion assays performed with 8 μm pore size membrane inserts.	Osteopontin overexpression enhanced the invasive ability significantly for LNCaP and somewhat for PC3. Anti-osteopontin antibodies in invasion assays significantly suppressed the response to osteopontin overexpression.
Conn [110]	PC-lo/diss, PC-hi/diss, HMVECs (human microvascular endothelial cells), CEFs (chick embryonic fibroblasts	3D	Intravasation	96-well clusters were coated with 5 μg/mL type I collagen, 10 μg/mL fibronectin, or 10 μg/mL Matrigel for adhesion assays. For invasion assays, cells were plated on 24-well clusters with 8 μm size pores with 2 μg/mL Matrigel.	Western blot and qPCR showed substantial reduction of epithelial marker E-cadherin in PC-hi/diss and demonstrated increased ability to invade through Matrigel compared to PC-lo/diss.
Zervantonakis [115]	HT1080 human fibrosarcoma, MDA231 breast carcinoma, primary MVEC, macrovascular endothelial cells. RAW264.7	3D	Intravasation	3D ECM hydrogel sandwiched between two microfluidic channels (500 μm wide, 20 mm length, and 120 μm height).	Permeability measurements indicated that signaling with macrophages, via secretion of tumor necrosis factor-α, degraded the endothelial barrier and increased intravasation rates.
Li [116]	MDA-MB-231, PC3, MDA-MB-435S, Raw 264.7 mouse macrophages.	3D	Intravasation	Collagen gel sandwiched between two microchannels (500 μm width) in PDMS devices.	The presence of macrophages enhanced the speed and persistence of PC3 cell migration in an MMP-dependent fashion.
Shin [119]	Metastatic LOVO, non-metastatic SW480	3D	Intravasation-Extravasation	Device consists of two parts; intravasation chamber for 3D culture using Matrigel, and extravasation chamber for detection of metastasized cells.	Invasion of LOVOs was higher compared to SW480. Treatment of cells with MMP inhibitors decreased invasion.
Cui [120]	Primary human vascular endothelial cells, MDA-MB-231, MCF-10A.	3D	Intravasation-Extravasation	Porous membrane (20 μm thickness, 4 mm by 4 mm area) sandwiched between two flow layers fabricated using soft lithography for PDMS. Underneath the layer is an accessible microchamber for cell collection.	Migratory MDA-MB-231 cells exhibited a higher body aspect ratio and faster planar migration compared to non-migratory cells.
Sima [123]	PC3	3D	Intravasation-Extravasation	L-shaped glass biochip fabricated by femtosecond laser-assisted etching. Two microreservoir inlets and one microreservoir for collecting migrated cells (1000 μm diameter, 280 μm height). Two embedded connecting channels (1500 μm length, 60 μm height). One channel consisted of two rows of glass elliptical pillars (150 μm diameter) 200 μm apart. Collagen coating.	PC3 cells migrated through narrow submicrometer channels with speeds up to 1.5 μm/min. The cells retained viability during and after migration, and the probability of proliferation was unchanged.
Stott [139]	Serum specimens from healthy and metastatic prostate cancer patients	3D	CTCs	CTC-Chip with a surface area of 970 mm^2^ and 78,000 microposts coated with antibodies to EpCAM. Image processing algorithms used with scoring criteria to analyze CTCs in a 3D matrix.	CTCs in blood circulation had a short half-life for patients with preoperative CTCs.
Kirby [140]	Peripheral blood samples from healthy and metastatic castrate-resistant prostate cancer patients	3D	CTCs	Oxygen plasma deep reactive ion etcher used to etch to a depth of 100 μm. PDMS sheets (3 mm thick) were clamped to the top of the device to create closed channels populated with post arrays.	Median of 54 CTCs/mL captured in CRPC patients versus 3 in healthy donors. Chip design allowed for monitoring of drug-target engagement.
Au [143]	Breast or melanoma patient liquid biopsies	3D	CTCs	Devices mimicked hydrodynamic properties of capillary networks (ΔP = 5–58 mm Hg) by tapering 16 parallel microchannels (50 μm diameter) into 5 μm, 7 μm, or 10 μm constrictions.	Clusters transited through capillaries in a single-file manner.
Vigmostad [144]	PC3, PrEC LH	3D	CTCs	Micropipette puller, microforge, micromanipulator, and thin-walled glass capillaries (5–8 μm internal diameter) used to partially aspirate a suspended cell and record measurements at various suction pressures.	PrEC LH cells were 140% stiffer than PC3 without exposure to FSS and experienced no significant change after FSS exposure. The Young’s modulus after exposure to high and low FSS increased for PC3.
Marcolino [148]	PNT2-C2, PC3, HuVEC	3D	Extravasation	In vitro extravasation assays performed using transwell chambers with Matrigel coating.	Endogenous PRH knockdown led to significant decrease in extravasation.
Manuelli [155]	Human prostate cancer cells (1532-CP2TX, 1535-CP1TX, 1542-CP3TX), AsPC-1 human pancreatic tumor cells, PC3.	3D	Extravasation	Coverslips were coated with Cy3-conjugated gelatine.	All cancer cell lines except for PC3 demonstrated the ability to synthesize invadopodia.
Lautscham [158]	A125 lung carcinoma, MDA-MB-231, HT-1080, primary breast cancer cells (IFDUC1)	3D	Extravasation	PDMS devices with a stiffness of 1.77 mPa and linear channel segments (20 μm length, 3.7 μm height, decreasing width from 11.2 to 1.7 μm) were used to measure the migratory ability of cells. Self-assembled collagen networks (550 Pa) with 3 μm pore size were used to investigate migratory behavior in a soft environment.	The nucleus was the limiting biophysical factor for 3D invasion. Cell stiffness was weakly correlated with invasiveness.
Gakhar [163]	PC3, C4-2, LNCaP, MDA PCa 2b, KGI	3D	CTCs, Extravasation	Microrenathane tubes (50 cm length, 300 μm diameter) were incubated with 10 μg/mL human recombinant IgG E-selectin and mounted on a microscope. Known concentrations of cells were perfused over the surface to measure interactions with E-selectin.	CTCs from prostate cancer patients tethered and interacted with E-selectin and E-selectin expressing HUVECs.
Chen [166]	GFP or RFP-expressing HUVEC, NHLF (normal human lung fibroblasts), MDA-MB-231, A-375 MA2, 4T1, SUM 159	3D	Extravasation	Three ECM hydrogel regions (1.3 mm width, 110 μm height, 0.8 cm length) separated by media channels and held in place via small trapezoidal microposts.	β1-depleted cells could not sustain protrusions into the matrix compared to control cells, suggesting β1 is a requirement for invasion through the endothelial basement membrane.
Verbruggen [169]	MLO-Y4 osteocyte-like mouse cell line, MDA-MB-231, MCF-7, PC3, LNCaP	3D	Extravasation	PDMS device containing two parallel microchannels separated by PDMS membrane (2% porosity) for cancer cell and osteocyte co-culture. Channels were coated with collagen I. Mechanical stimulation was generated using increased flow rate (1000 μL/h compared to 30 μL/h) in osteocyte channel.	Mechanical stimulation of osteocytes led to increased invasion of breast and prostate cancer cells and may prohibit the suppression of metastasis.
Shulby [173]	PC3 N, PC3 ML (bone metastatic human prostate carcinoma), LNCaP, hFOB (human osteoblasts), human MDA PCa 2b, DU145	3D	Bone tropism	Flow chamber cell adhesion assay performed using 24 × 50 mm glass coverslips coated with collagen and fibronectin. Cell migration assay performed by plating cells on FluoroBlock insert (8 μm pore size) in a 24-well plate.	Bone-derived prostate cancer cells adhered to bone marrow endothelial cells under flow.
Dimitroff [175]	Human HPC KG1a, murine monocytic WEHI-3, Human prostate tumor MDA PCa 2b, PC3, PC3M, PC3M Pro-4, PC3M LN-4, PC-R1, PC-E1, LNCaP, LNCaP Pro-5, LNCaP LN-3	3D	Bone tropism	Cell rolling assessed at 0.6 dynes/cm^2^ at 100× magnification using parallel-plate flow analysis. E-selectin/immunoglobulin-reactive 150 kDa membrane proteins analyzed using MALDI-TOF mass spectrometry.	PSGL-1 expression may be associated with bone metastasis. Bone metastasis of prostate cancer may mirror the process of HPC homing to bone.
Sottnik [177]	MLO-Y4 (murine osteocytes), DU145, LNCaP, PC3, BPH-1, H441, A549, MDA-MB-231, MCF7	3D	Bone tropism	Cells were seeded into Thermo Fisher Scientific Opticell cassettes. Hydrostatic pressure was adjusted using an IV bag containing cell culture media.	Applying pressure to osteocytes induced prostate cancer growth and invasion via the upregulation of CCL5 and MMP.
Hsiao [178]	PC3, HUVEC, MC3T3-E1	3D	Bone tropism	PDMS device with two stacked channels separated by semi-permeable 5 μm pore size polycarbonate membrane. Upper channel (200 μm height, 50 μm width) had 28 side channels (200 × 200 × 200 μm) and was used for cell capture. Lower channel (100 μm height, 2 mm width). Membrane surfaces treated with 1% *w*/*v* Pluronic F108.	Proliferation rate of PC3 was greatly decreased without a reduction in viability.
Bischel [179]	LNCaP, C4-2B, MC3T3, MC3T3-E1	3D	Bone tropism	ECM hydrogel in a microfluidic channel.	Bone metastatic LNCaPs (C4-2Bs) demonstrated increased invasive ability compared to LNCaP in co-culture with osteoblasts.
Ahn [180]	SW620 (colorectal cancer cells), MKN74 (gastric cancer cells), HUVEC	3D	Colorectal and gastric cancer metastasis to the bone	PDMS single-channel devices (800 μm width) were bonded to glass coverslips with ports for hydrogel injection and medium reservoirs.	High concentrations of hydroxyapatite drastically reduced migration of SW620 and MKN72.
Mei [181]	MLO-Y4, RAW264.7, MDA-MB-231, HUVEC	3D	Breast cancer metastasis to the bone	Double channel PDMS microfluidic device with fibronectin coating. Lumen channel was coated with Matrigel and collagen mixture. Custom microfluidic pump designed to operate in oscillating fashion.	Mechanical stimulation of osteocytes reduced breast cancer extravasation via reduction of extravasation distance and percentage of extravasated side-channels.
Jasuja [182]	hMSCs (PT-2501), PC3	3D	Bone tropism	Biocompatible crosslinked PMMA device with a rectangular culture chamber (50 mm × 30 mm × 32 mm) each supporting a scaffold sample were exposed to two flow rates (0.2 mL/min and 0.05 mL/min). A gas permeable membrane on top of the chamber permitted gas exchange with the culture chamber.	CXCR4 upregulation in bone led to a high migration rate of PC3.
Osawa [183]	HS5 (human bone stromal cells), PC3-GFP, C4-2B	3D	Bone tropism	6-well dish was used to create a recirculating system with primary tumor and metastatic sites. PE tubing coated with collagen I was used to connect the primary and metastatic sites to each other as well as to a peristaltic pump.	Macrofluidic model demonstrated cell migration from primary sites to metastatic sites.
Wheeler [195]	MDA-MB-231, MCF7, hepatocytes, NPC (neural progenitor cells)	3D	Dormancy and colonization	Scaffolds of commercially available LiverChip device (CN Bio Innovations Limited) were coated with 1% rat tail collagen type I and seeded with hepatocytes and NPCs.	Breast cancer cells integrated into hepatic niche and entered quiescence and therefore did not interfere with hepatocyte function.
Hassell [198]	H1975 human NSCLC adenocarcinoma cells, primary lung alveolar cells	3D	Dormancy and colonization	Bilayer PDMS microfluidic device (top channel: 1 mm width, 1 mm height; bottom channel: 1 mm width, 200 μm height) with a porous ECM-coated membrane (0.4 μm or 9 μm pore size). Cyclic suction was applied to two parallel side chambers to mimic physiological breathing motion.	Lung cancer cell growth and invasion were sensitive to cues associated with breathing motions.
Ma [199]	Human foreskin fibroblast cells, K-562, Jurkat cells	3D	Dormancy and colonization	PDMS microfluidic device with a unique droplet collection chamber design and filters to prevent debris. The channel height was comparable to that of a cell diameter.	Droplet microfluidic technology allowed for the characterization of metabolic differences between proliferating and quiescent cells.
Liu [201]	REF/E23 derived from rat embryonic fibroblasts REF52	3D	Dormancy and colonization	PDMS microfluidic devices bonded with glass slides. Channels were coated with 2% fibronectin.	Increasing flow rate as well as increased extracellular factor replacement drove cells to shallower quiescence.

## 4. Prostate Cancer Detection

Invasive biopsy, in which tissue or fluid is removed from the prostate for subsequent testing, is still the most reliable method for diagnosing prostate cancer, while the digital rectal exam (DRE) and prostate-specific antigen (PSA) blood test remain the cornerstones for screening with multiparametric magnetic resonance imaging (mpMRI) for local staging [202]. Several imaging methods have been adopted to scan for prostate cancer; all have certain advantages and disadvantages, and most are expensive and sophisticated with a lack of specificity and sensitivity [203]. Indeed, the lack of early detection, conventional diagnostic methods, expensive detection techniques, and unavailability of kits in developing countries have all hindered confronting prostate cancer worldwide. Naturally, non-invasive and cheap detection of biomarkers using liquid biopsy has received significant research and commercial attention to enable timely screening for prostate cancer [204]. Microfluidics, in particular, offers many promising benefits while meeting the demand for low-abundant blood cancer diagnosis [22], and it is envisioned to represent a paradigm shift in cancer health care [205].

### 4.1. Prostate Cancer Biomarkers

Early detection and regular monitoring of specific biomarkers at different stages of prostate cancer progression are important for screening. These biomarkers are directly linked with pathological, physiological, and pharmacological processes; they include specific cells, proteins, DNAs, RNAs, and biological analytes present in tissue, serum, blood, and urine samples. Early prostate cancer diagnoses could inform the follow-up control and treatment of the disease in efforts to maximize life expectancy with improved quality of life. A list of biomarkers currently used in clinics for assisting early diagnosis of prostate cancer has been compiled [203]; however, prostate cancer is heterogeneous with many phenotype variations, and the search for more reliable biomarkers is ongoing as, for example, the presence of TMPRSS2-ERG fusion gene was reported to indicate a more aggressive prostate cancer phenotype [206]. To address a global unmet need for rapid and cost-effective prognostic and diagnostic tools for use at the bedside, a tissue-based biomarker panel was introduced to assess the disease aggressiveness and invasive potential of prostate cancer [207]; live tumor samples were cultured in a microfluidic device, enabling automated imaging of biomarkers under conditions optimized for maintaining primary cancer cells within a simulated extracellular matrix. The results of a normal set of phenotypic biomarkers yielded secondary metrics, oncogenic potential (OP) and metastatic potential (MP), for distinguishing between benign and malignant histology and allowing prediction of stage as well as adverse pathology, e.g., extra-prostatic extension (EPE) and lympho-vascular invasion (LVI). Similarly, a microelectronic point-of-care metabolite biomarker measurement platform was developed and used for prostate cancer detection [208]. The point-of-care system performed colorimetric quantification of a chosen metabolite panel and, since many blood metabolites have been implicated in prostate cancer, only four were selected for a proof-of-concept: L-amino acid (LAA), glutamate, choline, and sarcosine. Despite the potential of such a microfluidic-based platform to improve the diagnostic accuracy, it is still envisioned to be carried out in conjunction with a PSA test.

#### 4.1.1. Prostate-Specific Antigen (PSA)

**Figure 4:**
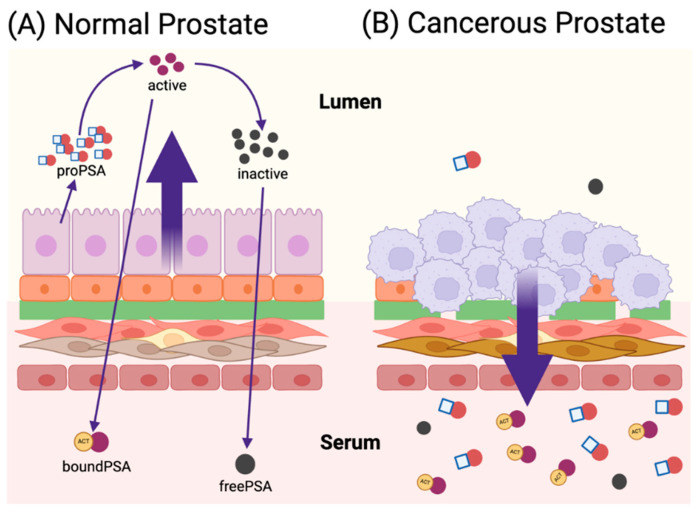
PSA in blood: (**A**) in a normal prostate, PSA is secreted as an inactive proenzyme (proPSA) into the lumen, where it is activated by cleavage of several amino acids. Active PSA entering the bloodstream intact is rapidly bound by protease inhibitors and circulates as bound PSA. A fraction of the active PSA also undergoes proteolysis in the lumen to generate inactive PSA, which can enter the bloodstream and circulates as free PSA. The cells are organized in a tight pattern, and only a very small amount leaks out of the lumen into the blood, resulting in a concentration of totalPSA (sum of freePSA and boundPSA) less than 4 ng/mL with a freePSA/totalPSA ratio higher than 25%. (**B**) In a cancerous prostate, both the cleavage activation and proteolytic inactivation in the semen are less efficient, resulting in relative increases in boundPSA and proPSA in the serum. The tumor cells are disorganized, and the layers in the microenvironment between the prostate gland and blood a blood vessel are disrupted, allowing much more PSA to leak into the bloodstream, leading to a totalPSA concentration greater than 10 ng/mL with a freePSA/totalPSA ratio lower than 25%. A PSA concentration between 4 and 10 ng/mL is considered a ‘gray zone’.

Prostate-specific antigen (PSA) is a protein normally secreted by both prostate epithelial (luminal) and cancer cells, as shown in Figure 4. PSA is an androgen-regulated serine protease whose biological role in semen is to hydrolyze the high-molecular-weight proteins secreted by the seminal vesicles; this converts the seminal gel to its fluid form, enabling the spermatozoa to swim free. While it is naturally present in semen, occasional molecules escape into the prostate-gland interstitium and, from there, via the lymphatics to the blood; therefore, the PSA concentration in blood is a million times lower than in semen [209]. Despite inherent limitations of PSA, it is still the most widely used oncologic biomarker reducing the proportion of patients diagnosed with advanced disease [210]. Detection of a high PSA level in a test may indicate the presence of prostate cancer; however, other conditions can also increase PSA levels and, thus, interpreting a high PSA score can be complicated. Nevertheless, close monitoring of elevated PSA levels could be decisive to confirm prostate cancer and, even for those already diagnosed with the disease, the PSA test may be used to evaluate treatment effectiveness or check for cancer recurrence [203]. Although PSA is consistently expressed in prostate cancer, its level of expression on a per-cell basis is lower than in normal prostate epithelium reflecting AR transcriptional activity [211]. PSA is secreted into the prostatic lumen as an inactive proenzyme (proPSA), where it is activated by cleavage of several amino acids. Active PSA entering the bloodstream intact is rapidly bound by protease inhibitors (primarily alpha1-antichymotrypsin) and circulates as bound PSA (Figure 4A), while a fraction of the active PSA undergoes proteolysis in the lumen to generate inactive PSA, which can enter the bloodstream and circulates as free PSA. Both the cleavage activation and proteolytic inactivation in the semen are less efficient in prostate cancer, leading to relative increases of bound PSA and proPSA in the serum [211]. Prostate cancer patients have a much higher total PSA (which is the sum of free and bound PSA) with a lower free-PSA and higher bound-PSA fraction (Figure 4B); thus, measurement of the ratio of free/total PSA (which is the sum of free and bound PSA) can significantly increase the sensitivity and specificity of prostate cancer detection. Tests are also under development to measure forms of proPSA, which may further enhance the ability to detect early-stage prostate cancer [212]. While it is generally accepted that PSA screening has led to a dramatic increase in the prostate cancer detection rate, the debate whether PSA screening significantly reduces mortality from prostate cancer is yet to be resolved [213]. Furthermore, the results of the test itself, utilizing a venous blood sample of to measure PSA concentration, could be affected by various factors, including the measurement technique [209]. PSA-based decision-making in prostate cancer indeed has significant shortcomings associated with the over-detection and over-treatment of clinically unimportant cases, resulting in an underwhelming impact on mortality [214]. However, as the PSA test remains a valuable tool in screening and monitoring of patients, the development of improved diagnostic methods may lead to new, efficient approaches for prostate cancer detection and treatment.

Microfluidic platforms have been developed as biosensors for measuring PSA concentration in biofluid samples, like in the standard PSA test, or as in vitro models for investigating PSA biology. For PSA concentration measurements in serum blood samples, commercial protein biochips were utilized to examine whether the hypothesis that pathologic states within the prostate could be reflected by changes in serum proteomic patterns [215]. The chips were designed with modified surface areas for the affinity-based selective binding and profiling of protein subsets from complex biological samples. Serum proteomic mass spectra were analyzed to reveal a pattern discriminating between patients without (PSA < 1 ng/mL) and with prostate cancer (PSA > 4 ng/mL), and the results indicated that serum proteomic patterns could be of value for whether to proceed with a biopsy on patients with elevated PSA levels. Adopting a similar approach, a rapid immunosensor was reported for detecting free and total prostate-specific antigen (f-PSA and t-PSA) tumor markers using a transducer coated with a pH-sensitive polymer film [216]. Nitrocellulose membrane strips were functionalized with f-PSA and t-PSA antibodies and used as supports for performing noncompetitive immunoassays. This one-step immunosensor exhibited high sensitivity to detect both forms of PSA and, considering its portability and robustness, it could be useful for early prostate cancer diagnosis. PSA detection based on a competitive immunoassay was also implemented in developing a microfluidic biosensor with an embedded three-electrode configuration to enhance its sensitivity for nanoliter-volume samples [217]. Following on-chip functionalization of the nanoelectrodes with a PSA complex, the minimum detection limit of PSA was about 10 pg/mL, paving the way to fabricate ultrasensitive bio-signal transducers in a chip-based analytical platform. Based on Electrochemical Impedance Spectroscopy (EIS), a microfluidic biosensing platform was developed to provide a diagnostic tool for label-free and sensitive analysis of the PSA test [218]. Two microelectrode arrays were functionalized with two different antibodies (Anti-freePSA and Anti-total PSA), and interactions with antigens were detected as a change in the impedance due to the formation of bound complexes. The measured signals from the two sensor arrays were used to estimate the ratio between the two antigen forms, particularly in the “gray zone” (4 ng/mL < PSA < 10 ng/mL), demonstrating the ability of the system to rapidly distinguish between conditions of PCa or BPH on the basis of the freePSA/total PSA ratio. Other microfluidic devices designed for PSA sensing include mass-sensitive cantilevers sensors [219] and immunosensors using surface plasmon resonance (SPR) with a quartz crystal microbalance (QCM) [220]. A common challenge to all affinity-based detection methods is the non-specific binding of non-target species present in the sample and, consequently, the interpretation of molecular recognition in biology has evolved from the key-and-lock to the allosteric concept in recognition of conformational transitions and the role of molecular ensembles [221].

Since in vitro prostate cancer research involved cell lines lacking predominant physiological features, such as the expression of pertinent biomarkers, microfluidic systems have also been designed to replicate the in vivo cancer microenvironments. An early in vitro human prostate cancer model was established by co-culturing prostate fibroblast and cancer cells (LNCaP) on microcarrier beads to evaluate androgen-induced growth and PSA expression [222]. The co-cultured fibroblasts and cancer cells responded to DHT-induced signals of growth and differentiation similar in vivo observations, highlighting the synergetic effects of androgen stimulation and stromal interaction. Recently, an MPS model of prostate cancer was presented for exploring PSA and microRNAs secretion of in vitro, where androgen-sensitive (LNCaP) and androgen-insensitive (PC3) cells were cultivated in standard and 3D cultures [223]. LNCaP cells maintained a constant PSA secretion and, in contrast, PC3 cells did not exhibit complex structures in the MPS model. At the gene level, PSA expression was upregulated in PC3-MPS and downregulated in LNCaP-MPS. The results not only revealed the sensitivity of gene expression level to external stimuli but also demonstrated the potential of using MPS models to study important phenotypical changes in prostate cancer cells.

#### 4.1.2. Circulating Tumor Cells (CTCs)

The release of CTCs is regarded as one of the early steps during tumor formation, and 40% of patients—who still have tumor-free lymph nodes—already have CTCs in their bloodstream. CTCs enumeration is an important component in cancer diagnostic, and their detection is currently included in international tumor staging systems to assist in predicting disease progression and monitoring treatment efficiency. Furthermore, metastatic cells often have different features than the primary tumor and, thus, an explicit characterization of CTCs is important for further application of clinical treatment [132]. The vast majority of CTCs are vulnerable to anoikis and immune attack and, consequently, are very rare with a short half-life time [224]. Despite their rarity, CTCs have been considered as a potential alternative to invasive biopsies as a source of tumor tissue for the detection, characterization, and monitoring of non-hematologic cancers [225]. The isolation of CTCs directly from blood as a real-time liquid biopsy has therefore received major attention since further analysis of these cells may greatly aid not only clinical applications but also basic research. Among the various approaches to isolating CTCs, microfluidics provides distinct advantages, such as low-cost, rapid detection, and precise control, as well as continuous sample processing and easy integration of various functions within a single device. Multiple reviews are now available providing summaries of the latest progress detecting and analyzing CTCs, as well as the malignant subset of circulating cancer stem cells (CCSCs), including discussions of fabrication technologies, device performance criteria, and future clinical applications of microfluidic CTC products for cancer diagnosis, including its relapse prediction [28,226,227,228]. Most data available on the CTCs role in cancer are based on randomized studies that do not translate into clinical practice, due, in part, to inconsistent reported results stemming from ambiguity in either CTC definition or performance criteria. While what biologically constitutes CTCs is clear, classifying captured cells from blood samples as CTCs is not straightforward; thus, in a definition gaining wider acceptance, CTCs are nucleated cells—a nucleus surrounded by cytoplasm with a positive cytokeratin (CK+) and a negative leukocyte antigen (CD45-). The clinical relevance of CTCs in prostate cancer has been highlighted as they emerge as a viable solution to the lack of tumor tissue availability for patients with a variety of solid tumors, offering attractive perspectives in disease management, including roles in prognostics of non-metastatic and metastatic tumors, monitoring treatment outcomes, and use as a surrogate marker for survival [229,230]. Several microfluidic technologies have been proposed to exploit biochemical or biophysical characteristics for isolating CTCs from complex samples [231]; each has advantages and disadvantages, especially techniques based on a single targeting criterion. Size-based filtration techniques are not sufficiently specific as there can be a significant overlap in size and other biophysical properties between target CTCs and non-target leukocytes [232], and affinity-based methods relying on binding between antibody ligands and target cell receptors are not very specific due to the same allosteric concept hindering affinity-based PSA detection [221]. Therefore, more sophisticated approaches incorporate at least two filtration criteria. For example, to isolate CTC clusters rather than individual CTCs, a two-stage microfluidic device was designed capitalizing on two geometric properties, size and asymmetry, as small cell clusters are probably asymmetrical and elongated, affecting the flow pattern and permitting isolation using vorticity [233]. In label-independent methodologies, CTCs were captured in a Parsortix cell separation system based on size and deformability [234]; and, utilizing optimal multi-parameter conditions, a microfluidic-elasto-filtration device was proposed, accounting for cell size, pore diameter, cell elasticity, and hydrodynamic drag force to obtain a critical elasto-capillary number as the filtration criterion [235].

The drive to introduce new microfluidic systems with superior performance in isolating CTCs from clinical blood samples has not abated. The CellSearch system was approved by the U.S. Food and Drug Administration (FDA) for breast and prostate cancer in 2004 and 2008, respectively; however, it was approved only for monitoring the metastatic disease, i.e., not for diagnosis, and its clinical application is still controversial. Thus, the research and development of CTC detection remain highly active, and only a few more recent examples are discussed. Three-dimensional printing technology was utilized to fabricate microfluidic devices containing certain interior structures to increase the surface area, which was functionalized with anti-EpCAM ligands; the high surface area was combined with fluid flow manipulation for optimal surface ligand–cell receptor binding to enhance the capture efficiency of EpCAM-expressing CTCs from bodily fluids [236]. The unique metabolic feature of all cancer cells characterized by high rate of glycolysis, rendering their surface charge negative, was utilized as a biophysical marker in a label-free technique for isolation of CTCs; super-paramagnetic positively charged nanoparticles (SPPCNs) were mixed in suspension bounding to the negatively charged CTCs, and the strong particle–cell binding then allowed for the efficient magnetic separation and isolation of CTCs from clinical blood samples, followed by immunofluorescence staining for detection of biomarkers expression [237]. A microfluidic chip, featuring an upstream channel for size-based filtration and a downstream chamber for cell trapping and culture area, was proposed as for on-chip streamlining cell separation, immunofluorescence assay and in situ culturing to minimize the risk of cell loss during CTC procurement [238].

Isolation of circulating cancer stem cells (CCSCs) can be even more challenging not only because of their scarcity but more so due to the complexity of the phenotype confirmation process and, to meet this challenge, an integrated combinatorial Raman-Active Nanoprobe (RAN) system was incorporated with a microfluidic device for processing blood samples. The nanoprobes featured unique collections of antibodies, double-stranded DNA linkers, and Raman dyes, having one of the nanoprobes functionalized with anti-CD133 ligands to specifically target CCSCs; thus, CTCs and CCSCs were isolated and classified based on the expression of their respective surface markers via specific RANs signals [239]. Isolated CCSCs were then employed for tumor phenotyping to characterize their tumorigenicity potential, with exclusion of peak overlapping in the CCSC analysis providing molecular characteristics of tumor subtypes. A microfluidic platform, consisting of microchannels functionalized with antibodies against either EpCAM or CD133 biomarkers, was similarly constructed to isolate CTCs and CCSCs from blood sample of cancer patients [240]. Isolated cells were confirmed via staining cytokeratin (CK) and CD45; in some cases, the complementary assessment of both CTCs and CCSCs appeared to be advantageous for assessing the tumor progression profile. EpCAM has been popularly used for targeting tumor cells circulating in the bloodstream; however, it has been shown that there are CTCs devoid of EpCAM expression, as well as non-CTCs expressing EpCAM in the blood of cancer patients [232]. Likewise, although CD133 function was linked to CCSCs biology, the underlying mechanisms involved in the CD133-mediated induction of stem-like properties in cancer cells have yet to be elucidated [240]. Consequently, while it was possible to isolate CTCs and CCSCs by targeting EpCAM and CD133, respectively, there was likely a population of these cells escaping capture due to low or no antigen expression.

To date, CellSearch is the only system validated by U.S. Food and Drug Administration (FDA) for CTC measurement in metastatic prostate cancer. Ferrofluid nanoparticles coated with EpCAM antibodies bind to EpCAM-expressing CTCs in this approach to facilitate their isolation by magnetic-activated cell sorting (MACS). Immunofluorescence (IF) staining is then utilized to enumerate CTCs as nucleated cells positive for cytokeratin 8, 18, or 19 and negative for CD45 expression [241]. Since CTCs isolated by various assays could be apoptotic or may lack stem cell properties, never giving rise to an overt metastasis, an epithelial immunospot (EPISPOT) technique was applied for detecting only viable CTCs. In this method, EpCAM−/+ tumor cells are enriched by depletion of CD45+ hematopoietic cells; the enriched CTCs are then cultured in vitro, allowing for a sufficient amount of secreted marker proteins to accumulate and form immunospots, as dying tumor cells do not secrete adequate protein amounts and thus are not detected [242]. As a proof of concept to isolate CTCs in vivo from blood of PCa patients, a medical wire (CellCollector) was used to isolate CTCs in blood samples of PCa patients; the gold-coated tip of the wire was covered by a polycarboxylate layer and, following activation, the wire tip was functionalized with the EpCAM antibodies to capture target CTCs for enumeration and molecular characterization [243]. The clinical relevance of blood tests for monitoring minimal residual disease (MRD) in non-metastatic prostate cancer was demonstrated by applying these three CTC assays (CellSearch, EPISPOT, and CellCollector) to screen for CTCs blood samples from prostate cancer patients before and after radical prostatectomy (RP). Considering the results of all three systems, CTCs were detected in 81.3% of the patients, suggesting that CTC-based liquid biopsy can potentially be used for monitoring MRD in non-metastatic prostate cancer patients [244]. Clinical validity of CTC detection as a biomarker for predicting overall survival of patients with metastatic castration-resistant prostate cancer patients (mCRPC) was provided by the application of the AdnaTest, in which magnetic beads conjugated with HER2 and EpCAM antibodies were utilized to isolate CTCs, followed by qRT-PCR analysis of captured cells for expression of prostate-specific genes (KLK3, PSMA, and EGFR) [245]; assays were also designed to detect AR splice variants and ligand-binding domain mutations associated with resistance to AR-directed therapies. An efficient strategy to isolate CTCs was reported using another immune-affinity approach, where a chemical ligand-exchange reaction was engineered to release cells captured on a gold nanoparticle (NPs) coating bound to anti-EpCAM functionalized surface of a herringbone microfluidic device (NP-^HB^CTC-Chip); a higher capture efficiency of prostate cancer cells (PC3) was observed, with lower nonspecific binding, as well as improved cell release efficiency and viability due to the inclusion of thiol-modified gold NPs [246]. CTCs were captured from blood of prostate cancer patients on a biofunctionalized substrate using a microfluidic probe integrated with herringbone structures for microvortex generation, and the bottom capture substrate of the herringbone microfluidic probe (HB-MFP) was functionalized with multiple biorecognition ligands; CTCs were captured efficiently from blood samples via specific EpCAM, PSMA, and PSA antigens revealing certain CTC phenotypes based on their expression levels [247]. CTC-based multigene models, using a microfluidic system, were evaluated as a diagnostic tool for distinguishing between healthy controls and patients with either LPCa, metastatic hormone-sensitive prostate cancer (mHSPC), or mCRPC; CTCs in each blood sample were first labeled by magnetic nanobeads conjugated with EpCAM antibodies, followed by injection into a microchannel where the magnetically labeled CTCs were pulled laterally toward their outlet along slanted ferromagnetic wires due to a high-gradient magnetic field around the wires [248]. The expression profiles of CTCs collected from the magnetophoretic microseparator were analyzed, indicating that the CTC count increased at later stages of prostate cancer in correlation with serum PSA levels and, furthermore, the CTC-based genetic information suggested that prostate cancer progression was related to the expression of the prostate- and epithelial-specific genes. Finally, in an effort to circumvent the dependency of CTCs isolation on EpCAM expression altogether, CD45 was identified as an ideal target for removal of white blood cells (WBCs) using negative MACS modality. In the updated CTC-iChip 2, size-based filtration separating nucleated cells from blood was combined with negative selection removing bead-conjugated WBCs, resulting in isolation of CTCs with greater reliability and throughput [232].

#### 4.1.3. Extracellular Vesicles (EVs)

In multicellular organisms, various pathways have evolved to control all aspects of cell physiology and intercellular communication, in which extracellular vesicles (EVs) are very significant. Three groups of EVs have been distinguished based on their size and biogenesis: apoptotic vesicles, microvesicles, and exosomes. Apoptotic vesicles are the largest, 500–5000 nm in size, originating from the outer membrane of an apoptotic cell and carrying a large amount of phosphatidylserine, and microvesicles, 100–1000 nm in diameter, emanate from the plasma membrane with an enriched cholesterol and phosphatidylserine structure. Exosomes are nanoscale membrane vesicles, 30–100 nm in size, formed by the endocytosis, fusion, and efflux of cells containing essential biomolecules such as lipids, proteins, mRNAs, and micro RNAs; they are actively released by most cell types into their extracellular environment, and as they can be isolated from different body fluids, several microfluidic strategies were reported for their isolation and analysis [249]. Exosomes have attracted attention due to their promising roles as both circulating disease biomarkers and therapeutic drug carriers. Tumor-derived exosomes contain various molecular constituents, which are critically involved in tumor initiation and progression reflecting the pathological and physiological state of parental tumor cells.

Given the obstacles encountered in CTC isolation and analysis, tumor-derived exosomes are gaining popularity as biomarkers for liquid biopsy to diagnose numerous cancers. Accordingly, various microfluidic platforms suitable for the isolation and detection of exosomes were described along with their performance criteria in terms of yield, sensitivity, and duration [250,251] and, more recently, selected microfluidic systems to detect EVs for cancer screening were compared and classified based on their size- or immunoaffinity-separation technique [252]. As the clinical translation of exosomes remains challenging due to their relatively low concentration in body fluids, microfluidic surface-enhanced Raman spectroscopy (SERS) is gaining significant attention as a label-free and sensitive technique for exosome isolation and characterization [253]. Combining on-chip continuous flow mixing with immunomagnetic isolation, an in situ Raman assay was developed to capture exosomes using anti-CD63 conjugated magnetic nanoparticles in microchannels with staggered triangular pillar array; the enriched exosomes were magnetically fixed on the Raman detection segment, and EpCAM-functionalized Raman beads with high nitrile densities were used as the probe to quantitatively detect exosomes from PCa cells (LNCaP) and healthy prostate cells (PrEC) by monitoring the intensity of the SERS peak [254]. The biochip was applied for the analysis of clinical serum samples, correctly distinguishing between PCa patients and healthy donors, thus demonstrating its potential as a clinical exosome analysis tool for the diagnosis of prostate cancer. Thus far, two liquid biopsy tests were approved by the FDA for detecting gene mutations in circulating cell-free DNA (cfDNA), Guardant360 CDx and FoundationOne Liquid CDx, and both are approved for patients with any solid cancer (e.g., prostate, breast). While the FDA has approved other blood tests that check for the presence a single gene mutation in tumor DNA, these are the first approved blood tests that check for multiple cancer-related genetic changes; the FDA approval only validates that the results of these blood-based tumor profiling tests can be used to guide the selection of targeted therapies. In prostate cancer, FoundationOne Liquid CDx is indicated for use as a companion diagnostic for four FDA-approved precision therapies, including an indication for Rubraca (rucaparib), a PARP inhibitor approved for the treatment of metastatic castration-resistant prostate cancer patients with BRCA1/2 mutations.

#### 4.1.4. Urine

Clinical analysis of urine has commonly been used to diagnose several diseases since it is a non-invasive, inexpensive, and fast diagnostic tool in health care. Changes in urine composition—such as changes in the levels of glucose, albumin, creatinine, and uric acid—may provide information about organ dysfunctions, drug abuse, and exposure to chemicals and toxins [255]. Although the application of microfluidics in urological research is scant, there is an upward trend in reports on microfluidic devices utilized in urinary analysis to provide high-throughput, high-precision, and low-cost analytical tools for sperm sorting, the detection of prostate and bladder cancer, as well as the characterization of cancer microenvironments [26]. It has further been envisioned that nano-diagnostic approaches can be integrated to discover new urinary biomarkers, specifically for precision prostate cancer diagnosis [256].

Prostate cancer gene 3 (PCA3), which encodes a prostate-specific mRNA, was considered as a promising diagnostic marker, and a prototype quantitative PCA3-based test for whole urine was characterized, with clinical specimen tests yielding specificity greater than that for the serum PSA assay [257]. Tumor exosomes isolated from the urine of patients were found to carry genetic information specific for prostate cancer and, as a proof of principle, they were used to detect two PCa mRNA biomarkers, PCA3 and TMPRSS2:ERG; furthermore, this noninvasive transcriptome analysis was also reported to be informative as to the overall tumor malignancy status [258]. Considering 8-hydroxy-2′-deoxyguanosine (8-OHdG) as an abundant product of DNA oxidation, a microfluidic system was used to detect this compound in human urine for estimating the oxidative DNA damage due to oxidation stress; the isolation of 8-OHdG from biological samples of prostate cancer patients was optimized using antibody-functionalized paramagnetic particles and a glassy carbon working-electrode serving as an electrochemical detector [259]. As PSA levels are elevated in both BPH and PCa, non-invasive urinary biomarkers were searched to distinguish between these benign and malignant prostate diseases; the presence of EGF, HE-4, and COL1A1 was analyzed and validated by ELISA, revealing that COL1A1 was significantly elevated in the urine of patients diagnosed with early or localized PCa versus BPH [260]. Elevated levels of several proteases were identified, using prostate tissue microarrays (TMAs), and substrate gel electrophoresis revealed elevated activity of both MMP-9 and MMP-2, suggesting that both may participate in the cleavage of collagen type 1, resulting in elevated levels of COL1A1 in the urine of PCa patients. A spiral microfluidic chip was developed for isolating tumor cells from the urine of patients with localized forms of PCa to provide a non-invasive and patient-friendly alternative, since only a small number of cancer cells shed into the bloodstream, perhaps requiring a large volume of blood for detection; the filtration principle was essentially size-dependent, relying on smaller hematologic components to be skewed toward the outer wall and the larger tumor cells to concentrate near the inner wall of a spiral microchannel with a trapezoidal cross-section [261]. Glypican-1 (GPC-1) was selected as the biomarker for identifying collected tumor cells, and the microfluidic device proved to be functional in capturing GPC1+ putative tumor cells; moreover, the number of captured GPC1+ cells was found to be correlated with the Gleason score used in grading prostate cancer. Due to its clinical importance as a biomarker for CRPC, and resistance to anti-androgen therapy, a liquid biopsy method was reported for analyzing androgen-receptor splice variant 7 (AR-V7) in the RNA of urine-derived EVs isolated by a lab-on-a-disc; rapid enrichment of EVs from urine samples was followed by mRNA extraction to allow for the measurement of both AR-V7 and androgen receptor full-length (AR-FL) mRNA levels by droplet digital polymerase chain reaction (ddPCR) [262]. Lower AR-FL and higher AR-V7 expression were observed in EVs derived from the urine of CRPC compared to HSPC patients, and the AR-V7/AR-FL ratio was higher in the urine EVs of advanced prostate cancer patients. To explore microRNA diagnostic potential, a panel of 12 PCa-associated microRNAs in urinary samples from patients undergoing biopsy was analyzed [263]. Receiver operating characteristic (ROC) curve results indicated that miR-16 and miR-195 are best candidates for urine-based prostate cancer detection. While PSAD exhibited some diagnostic value, with serum PSA displaying none, the combination of the two best microRNAs with PSAD improved the diagnostic power in the tested cohort, underscoring urinary microRNAs’ potential as clinically relevant biomarkers.

Identification of prostate cancer cells from voided urine in a microfluidic device was explored, integrating prostate-specific membrane antigen (PSMA)-based immunocapture with hexaminolevulinate (HAL)-based photodetection, for providing an accurate noninvasive alternative to current diagnostic methods. The device consisted of a test channel functionalized with anti-PSMA ligands, a positive-control channel coated with a polyoxazoline (POX) thin film for binding all cell types, and a negative-control channel with skim milk proteins blocking the POX-coating [264]. Stained prostate cancer cells spiked in urine samples were captured using HAL as a cancer-specific photosensitizer to avoid pre-staining. Optimum HAL incubation conditions were identified, where the mean HAL-induced fluorescence intensity of LNCaP cancer cells was three times that of PNT2 normal cells, providing an independent selection criterion, and the combination of anti-PSMA immunocapture with HAL-induced fluorescent detection enabled isolation of cancer cells with high sensitivity and specificity. The diagnostic performance of another microfluidic-based platform was evaluate using voided urine samples collected without prior digital rectal examination (DRE), where the applied biotechnology combined the photodynamic-diagnostic principle with immunocapture for prostate cancer detection from malignant cells shed in urine [265]. The functionality and sensitivity of the constructed platform were validated using both cultured cells and PCa patient urine samples. The microfluidic detection limit was confirmed by quantitative reverse-transcriptase polymerase chain reaction (qRT-PCR), successfully validating the presence of a PCa biomarker in the urine of cancer patients without prior DRE, and its performance (sensitivity and specificity) was in agreement with qRT-PCR data, encouraging the further development of noninvasive prostate cancer diagnostic technologies that do not require DRE.

## 5. Prostate Cancer Therapy

Surgery and radiotherapy have been the first-line treatment in localized disease, whereas hormone and chemotherapy are standard clinical treatments in recurrent or metastatic disease; but, some patients develop castration-resistance with poor prognosis and limited treatment options [266]. Since cultured cells often do not retain their original organ functions and morphologies in traditional culture systems, it is still difficult to predict drug efficacy, toxicity, and organs interactions. Reciprocal signaling between prostate stroma and its epithelium influences many aspects of prostate cancer pathogenesis, including the response and resistance to anticancer therapeutics, such as genotoxic drugs or engineered antibodies [267]. The tumor microenvironment is complex and dynamic, actively contributing to modifying tumor cell phenotypes involved in metastatic progression and treatment resistance. While reductionist experimental models may allow for the mechanistic understanding of the interplay between tumor cells and key cell types or compounds comprising the prostate stroma, the complexity of the combined stromal components indicates that system-based approaches may be required to fully understand how perturbations ultimately influence prostate cancer evolution. Aside from its contributions in prostate cancer development and detection, microfluidic technology has also been applied for exploring therapeutic aspects such as drug development and disease management [268]. Microfluidic-based ex vivo models of human organs, replicating organ-level functions amenable for long-term maintenance, are indeed poised to gradually bridge the gap between in vitro cell cultures and in vivo animal models [269]. Respiratory medicine, for example, likely faces a low number of new approved therapies, partly due to lack of models reproducing pathophysiological responses of human lung, paving the way for applying organ-on-a-chip technology—modeling human lung alveoli and small airways—not only in the initial stages of the drug discovery process but also in preclinical drug efficacy testing [270]; and a similar development is transpiring in prostate cancer.

### 5.1. Drug Discovery and Screening

The development of new compounds to treat human diseases, including prostate cancer, has been a stumbling block in the healthcare arena. Although animal models have been indispensable for preclinical drug screening, species differences remain a major obstacle and, consequently, assays based on human-derived cells have been actively pursued. For example, elucidating the androgen receptor (AR) structure and activity, along with its actions in cancer cell lines, led to rational drug design for treating prostate cancer, where drugs preventing androgen production and/or blocking AR action inhibit the growth of prostate cancer [271]. However, resistance to these drugs often occurs after a few years as the patients develop castration-resistant prostate cancer (CRPC), with a functional AR remaining a key regulator. The migration of PC3 within a collagen matrix was monitored using time-lapse video microscopy to investigate induction of a metastatogenic tumor cell type by neurotransmitters and its inhibition by established drugs [272]. Several neurotransmitters were found to induce tumor cell migration, activating the cyclic adenosine-monophosphate response element binding protein (CREB), and microarray analysis revealed changes of gene expression toward a highly motile tumor cell type, including the upregulation of the α2 integrin and downregulation of the tumor suppressor gene gelsolin. The induced migration was inhibited using clinically established antagonists to several receptors, such as β1- and β2-adrenoceptors, suggesting that well-established pharmaceutical agents such as β-blockers could also be used for the chemoprevention of metastasis. Combinational chemotherapy was performed on PC3 cells in a microfluidic high throughput drug screening (HTDS) system consisting of addressable cell culture chambers, where sequential combinatorial concentrations of two different drugs were generated by two microfluidic diffusive mixers; each diffusive mixer was connected to media and drug reservoirs via two integrated micropumps to generate programmable combinations [273]. Since curcumin was reported to inhibit NF-κB activation in prostate cancer cells by sensitizing them to TNF-related apoptosis-inducing ligand (TRAIL), the response of PC3 cells to combinational treatments of curcumin as a sensitizer for TRAIL-induced cell death was examined, demonstrating the utility of the microfluidic system. The optimal concentration of curcumin for sensitizing PC3 cells at lower-dose TRAIL treatment was determined, highlighting the system potential application for screening and optimizing drug combinations with small amounts of reagents in combinatorial chemotherapy against cancer cells.

The recent performance assessment and economic analysis of a human liver chip for predictive toxicology have so far provided the most compelling support for MPSs potential impact on the pharmaceutical industry. As current preclinical models often miss drug toxicities, causing pharmaceutical companies considerable waste of time and resources to develop drugs destined to fail, the systemic and quantitative evaluation of organ-on-a-chip technology predictive value was conducted. Human liver chips were tested for predicting liver injury caused by small-molecule drugs, and an economic analysis was performed to quantify their value if they were utilized in toxicity-related preclinical trials [274]. The liver chips were found to meet the qualification criteria across a set of known drugs with high sensitivity and specificity, and this performance level was estimated to annually generate over USD 3 billion for the pharmaceutical industry through increased productivity. These results decisively demonstrate how incorporating predictive microfluidic-based systems into workflows of drug development can significantly improve drug discovery, allowing the delivery of more effective medications to patients in shorter time at a lower cost.

### 5.2. Prostate Cancer Resistance to Treatment and Management

Androgen deprivation therapy (ADT) for treating metastatic prostate cancer patients is effective only initially and, therefore, secondary hormonal therapies are examined for suppressing AR reactivation in CRPC, requiring reliable biomarkers to guide their application since responses to AR pathway inhibitors vary significantly. AR signaling readouts of CTCs captured in a microfluidic system were measured before and after therapeutic interventions, revealing a predominant “AR-on” signature in CTCs of untreated patients whereas, in CTCs of CRPC patients, the signature was heterogeneous (“AR-on, -off, and -mixed”) [275]. ADT treatment was found to induce a profound switch from “AR-on” to “AR-off”, with secondary hormonal therapy in CRPC resulting in a varied response, while an increasing “AR-on” signature was associated with an adverse treatment outcome despite treatment with an androgen biosynthesis inhibitor; thus, the measurement of pre- and post-treatment AR signaling within captured CTCs may help target patients most likely to respond to second-line therapies.

The effects of hypoxia, a known prominent characteristic of the tumor microenvironment, on drug susceptibility were elucidated utilizing a microfluidic system; prostate cancer cells (PC3) were cultured in a vacuum-actuated microfluidic device and treated with varying concentrations of a chemotherapeutic agent (staurosporine) under normoxic and hypoxic conditions, while cell apoptosis was assayed using fluorescent probes [276]. For hypoxic experiments, the chip was placed in a hypoxia chamber and preconditioned at less than 1% oxygen before exposing the cells to staurosporine. Cells treated with staurosporine were dramatically less apoptotic under hypoxic compared to normoxic conditions, and resistance to the drug increased within an hour of hypoxic preconditioning. Moreover, cell apoptosis correlated with drug dose, although in each concentration, hypoxia reduced the apoptotic fraction significantly. Given the rapid nature of cell adaptation to hypoxia, this microfluidic-based approach offers significant advantages in assessing therapeutic agents under hypoxic environments and can be used to identify compounds inducing rapid apoptosis of hypoxic tumor cells.

Blood-based biomarkers, pursued mainly for early detection, can also be used to guide prostate cancer therapies, such as the curative treatment of localized and application of targeted agents in metastatic disease; they are particularly critical in metastatic prostate cancer, where bone metastases are not readily sampled. Thus, to analyze prostate CTCs, a sensitive and high-throughput strategy was established using microfluidic cell enrichment followed by digital quantitation of prostate-derived transcripts [277]. Digital RNA-PCR was applied to simultaneously detect tumor burden and lineage-specific CTC signaling pathways from CTC-iChip-enriched blood samples of prostate cancer patients. The digital quantitation of CTC-specific transcripts—enabling noninvasive monitoring—was found to predict drug response in metastatic cancer, as well as early dissemination in localized cancer and, therefore, could guide treatment selection for localized and metastatic prostate cancer.

Summary of selected microfluidic systems used in prostate cancer research (**Table 1.** continued).

**Reference**

**Cells/Samples Used**

**Culture Type**

**Field of**

**Investigation**

**Device Properties**

**Findings**
Annese [208]Plasma samples from healthy men and men with prostate cancer3DDetection of prostate cancer (PSA)Disposable chip cartridge (120 PGA chip package, CMOS chip, microfluidic capillary network, and reagents), reader (STM32F334R8T6 microcontroller on an ST Nucleo F334R8 board, 8.5 × 7.5 × 4.0 cm, 150 g weight), and GUI (custom software)System could detect prostate cancer with a sensitivity of 94% and specificity of 70%.Petricoin [215]Serum samples from: asymptomatic men aged ≥50 years; normal healthy men; men undergoing radical prostatectomy, (pre- and postoperative);2DDetection of prostate cancer (PSA, serum proteomics)Thawed sera were applied to a C16 hydrophobic interaction protein chip. SELDI-TOF serum profiling and bioinformatics algorithm used to analyze samples and distinguish between cancerous and benign prostate conditions over a range of PSA levels.Serum proteomics could distinguish prostate cancer with 95% sensitivity, even when PSA levels were in an indeterminate range (4–10 ng/mL). Test had a high clinical relevance as a complement to physical exams, imaging, and serum PSA levels.Fernández-Sánchez [216]f-PSA and t-PSA stock solutions2DDetection of prostate cancer (PSA)Integrated single-use immunosensor with nitrocellulose membrane coated with f-PSA and t-PSA antibodies. Storage blister with urea solution allowed for washing away unbound species. Impedance spectra were used to detect the specific affinity event.PSA concentrations were detected down to 3 ng/mL and allowed for visual detection via colloidal gold antibody tracers. Triroj [217]PSA in complex with GOx enzyme2DDetection of prostate cancer (PSA)Biochemical sensing platform with a working electrode (5 × 5 array of <100 nm pores) milled using an ion beam and I_2_ gas, PDMS microchannels (30 μm height), and microelectrode platform. Nanoelectrodes were functionalized to detect PSA-GOx complex via cyclic voltammetry.PSA concentration could be detected as low as 10 pg/mL. Chiriacò [218]Prepared solutions with varying PSA ratios2DDetection of prostate cancer (PSA)PDMS biochip with a microfluidic module (7 mm × 4 mm × 100μm height) and two transducer arrays of gold interdigitated microelectrode sensing platforms. Electrodes were functionalized to calibrate for f-PSA and PSA in complex with ACT.PSA ratios could be calculated to identify cases of prostate cancer or BPH with distinction.Zhau [222]LNCaP, normal human prostatic fibroblasts3DDetection of prostate cancer (PSA)Slow-Turning Lateral Vessel (STLV) seeded with Cytodex-3 microcarriers at a density of 5 mg/mL. Vessels were rotated at 25–30 rpm.DHT induced differentiation-promoting behavior of LNCaP in microgravity-simulated conditions. LNCaP expressed high levels of PSA mRNA and protein under coculture with fibroblasts.Kuske [244]Samples from 86 prostate cancer patients (median age 67) prior to radical prostatectomy2DDetection of prostate cancer (CTCs)Combined analysis from three CTC assays (CellSearch, CellCollector, and EPISPOT).CTC detection by EPISPOT prior to radical prostatectomy significantly correlated with PSA serum values.Gila [247]PC3, MCF-7, blood samples from patients with localized and metastatic prostate cancer3DDetection of prostate cancer (CTCs)Herringbone–microfluidic channel-less probe with EpCAM, PSMA, and PSA captured antibody stripes. Blood is injected from the central aperture and aspirated from crescent-shaped peripheral apertures.CTCs were captured from blood samples as they traversed several antibody capture lines.Cho [248]Peripheral blood samples from patients with localized prostate cancer, mCRPC, or mHSPC3DDetection of prostate cancer (CTCs)Microseparater with disposable substrate, two inlets and outlets, vacuum trench, and reusable substrate with ferromagnetic wires.Successful microfluidic isolation of CTCs indicated increased CTC count with progressing stages of prostate cancer.Wang [254]Exosomes extracted from LNCaP and PrEC (enriched with anti-CD63 magnetic nanoparticles), serum samples from 10 prostate cancer patients and 8 healthy cases2DDetection of prostate cancer (extracellular vesicles)PDMS devices (SU8-2075 negative photoresist) bonded to glass slides. Raman spectra were collected on a confocal micro-Raman system.Exosomes from clinical samples could be analyzed within 1 h at a limit of 1.6 × 10^2^ particles/mL with 20 μm samples.Rzhevskiy [261]DU145, midstream urine samples from 14 healthy patients and 14 patients with localized prostate cancer3DDetection of prostate cancer (urine)PDMS spiral microchannel device bonded to PDMS. Anti-Glypican-1 used as the primary antibody for identification of collected tumor cells.Device could capture DU145 cells with >85% efficiency and was functional in 79% of cases for capturing cells from urine samples of patients with localized prostate cancer.Borkowetz [263]PNT2 human normal prostate epithelium, LNCaP clone FGC and 11Rv13DDetection of prostate cancer (urine)Three microchannels as a positive control (biocompatible with PPOx), negative control, and test (limit non-specific binding between surface-bound PSMA antibodies) channels.Biosensing platform was able to identify and capture cells with a sensitivity and specificity of 72.4% and 71.4%, respectively.Chan [265]PNT2, LNCaP clone FGC, 22rv1, prostate cancer patient urine samples3DDetection of prostate cancer (urine)PMMA microfluidic channels coated with polyoxazoline.Photodynamic diagnostic with immunocapture could detect prostate cancer biomarkers without prior DRE with a sensitivity of 72.4% and specificity of 71.4%Lang [272]MDA-MB-468, PC33DProstate cancer therapyMixed cells with buffered collagen solution, neurotransmitters, and pharmacological substances. Cell–collagen mixture was filled into self-constructed migration chambers. Selected 30 cells in each sample and recorded migration every 15 min for 12 h.Strong increase in migratory activity of PC3 cells as a result of norepinephrine treatment. β1-specific blocker atenolol partially reduced migration; β2-specific blocker ICI 118,771 entirely inhibited norepinephrine induction.An [273]PC33DProstate cancer therapyPDMS device consisting of two layers. The first is a fluidic layer with a diffusive mixer and microchambers (100μL volume) for cell culture. The second is a pneumatic layer with 10 channels to control microvalves in the fluidic layer. The optimal concentration of curcumin for sensitizing PC3 cells at lower-dose TRAIL treatment was determined.

## 6. Summary

**Figure 5:**
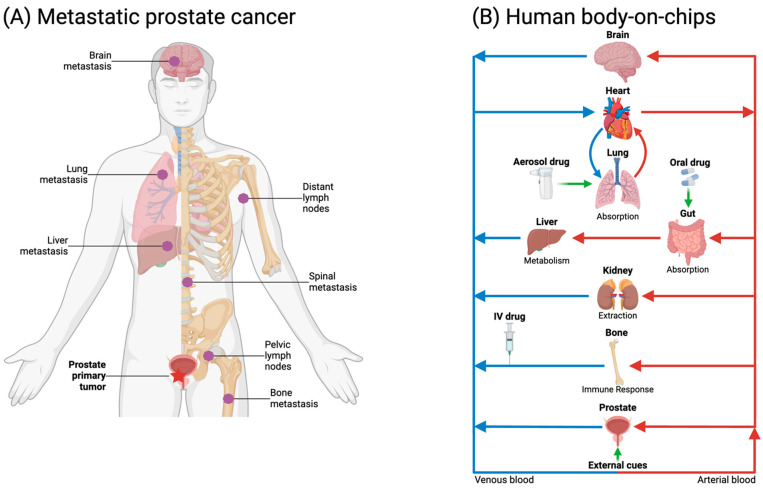
Microfluidic-based modeling of metastatic prostate cancer: (**A**) Prostate cancer usually spreads to the bones (spine) at the onset of metastatic disease and, at later stages, to distant lymph nodes, as well as the lung, liver, and brain. (**B**) A schematic diagram of fluidically linked multi-organ chips to mimic the physiological interactions among organs in the human body for prostate cancer research and development; aerosolized, oral, and intravenous (IV) delivery of drugs can be modeled by introducing them into the lung chip, the gut chip, or the vascular channel, respectively, and by linking the prostate chip as well, it will be possible to introduce external biochemical and biophysical insults encountered in prostate cancer tumor initiation and progression.

If prostate cancer metastasizes, it usually spreads to the bones (spine) as presented by the majority of patients. At later stages, the proportion of patients with atypical metastases is not unimportant, and the most common metastatic sites besides the bones are the distant lymph nodes, lung, liver, and brain (Figure 5A). These prostate cancer tumors, like other types of cancer, are not necessarily life-threatening as they may recede into dormancy. Metastatic prostate cancer though can turn deadly upon tumors re-awakening, with the transition from micro- to macro-metastases, resulting in the colonization and failure of secondary organs. In particular, metastatic castration-resistant prostate cancer (mCRPC) is still a daunting clinical hurdle since standard hormone therapy is no longer effective, requiring additional aggressive treatment to curb the growth of metastases. One in eight men diagnosed with prostate cancer is currently estimated to die of it and, for mCRPC patients, the 5-year survival rate is about 30%. Consequently, research efforts continue unabated to advance our understanding of how to prevent, detect, and treat prostate cancer, which require model systems for carrying out such studies. Given the limited value of animal and conventional cell culture models, failing to recapitulate the dynamic tumor microenvironment, microfluidic-based models are taking center stage as tools for prostate cancer research. Microfluidics is a rapidly evolving field at the intersection of engineering, physics, chemistry, and biology, which has been projected to revolutionize numerous aspects of the life science, biotechnology, and healthcare industries. Microfluidic technology offers unprecedented control and precision to manipulate minute volumes of fluids on the microscale for a wide range of applications, including cancer. Cellular co-cultures in microfluidic devices under media flow have already been utilized to reconstitute the critical functions of numerous human organs with high fidelity. However, the human body is very complicated, and individual organs-on-chips cannot faithfully represent human organs without accounting for the interactions between them and other systems within the body. Indeed, multiple organs-on-chips have been fluidically coupled into various systems, recapitulating whole-body inter-organ physiology to model complex human diseases [278]. Inspired by this progress, it is conceivable to incorporate, in the near future, a prostate-on-a-chip with patient-derived cells in a certain human body-on-chips format, as shown in Figure 5B, specifically for prostate cancer applications. Such a system will enable physiologically and clinically relevant studies of tumorigenesis by the in situ induction of genetic mutations in normal cells; pre-metastatic niche by monitoring organ response to tumor formation; the metastasis cascade from invasion to extravasation, including organotropism; dormancy and colonization, leading to relapse; biomarker discovery for early detection; and the prediction of responses to various therapies. Microfluidics is proving to be an important contributor for basic research, studying fundamental biophysical and biochemical mechanisms associated with prostate cancer development, as well as for clinical practices toward personalized precision medicine to eradicate prostate cancer.
